# Urinary proteome of dogs with kidney injury during babesiosis

**DOI:** 10.1186/s12917-019-2194-0

**Published:** 2019-12-04

**Authors:** D. Winiarczyk, K. Michalak, L. Adaszek, M. Winiarczyk, S. Winiarczyk

**Affiliations:** 1 Department and Clinic of Animal Internal Diseases, University of Life Sciences, Głęboka 30, 20–612 Lublin, Poland; 2 Department of Epizootiology and Clinic of Infectious Diseases, University of Life Sciences, Głęboka 30, 20– 612 Lublin, Poland; 3 Department of Vitreoretinal Surgery, Medical University of Lublin, Chmielna 1, 20–079 Lublin, Poland

**Keywords:** Acute kidney injury, Babesiosis, Dog, Proteomics, Urine

## Abstract

**Background:**

Acute kidney injury is the most frequent complication of babesiosis in dogs and may provide a natural model for identifying early and specific markers of kidney injury in this species. There are limited data on urine proteomics in dogs, and none of the effect of babesiosis on the urine proteome. This study aimed to identify urinary proteins of dogs with kidney injury during the natural course of babesiosis caused by Babesia canis, and to compare them with proteins in a control group to reveal any potential biomarkers predicting renal injury before the presence of azotemia.

Urine samples were collected from 10 dogs of various breeds and sex with naturally occurring babesiosis, and 10 healthy dogs. Pooled urine samples from both groups were separated by 2D (two-dimensional) electrophoresis, followed by protein identification using MALDI-TOF (matrix-assisted laser desorption ionization time of flight) mass spectrometry.

**Results:**

In total, 176 proteins were identified in the urine samples from healthy dogs, and 403 proteins were identified in the urine samples from dogs with babesiosis. Of the 176 proteins, 146 were assigned exclusively to healthy dogs, and 373 of the 403 proteins were assigned exclusively to dogs with babesiosis; 30 proteins were common for both groups. Characteristic analysis of 373 proteins found in dogs with babesiosis led to the isolation of 8 proteins associated with 10 metabolic pathways involved in immune and inflammatory responses.

**Conclusions:**

It was hypothesized that epithelial-mesenchymal transition might play an important role in the mechanisms underlying pathological changes in renal tissue during babesiosis, as indicated by a causal relationship network built by combining 5 of the 10 selected metabolic pathways, and 4 of the 8 proteins associated with these pathways; this network included cadherins, gonadotropin releasing hormone receptors, inflammatory responses mediated by chemokine and cytokine signalling pathways, integrins, interleukins, and TGF-β (transforming growth factor β) pathways. Those pathways were linked by interleukin-13, bone morphogenetic protein 7, α2(1) collagen, and tyrosine protein kinase Fer, which are potential biomarkers of damage during babesiosis in dogs, that might indicate early renal injury.

## Background

After heart failure, kidney disease is the most frequent cause of reduced quality of life and shortened survival of people and dogs [1–4]. Two different forms of kidney disease, acute kidney injury (AKI) and chronic kidney disease (CKD), are caused by various factors. In humans, 7.8% of patients with AKI develop CKD, and 4.9% of patients progress to end-stage renal disease [1]. Most AKI cases in medicine and veterinary science are diagnosed based on serum or plasma concentrations of non-protein nitrogenous creatinine (Cr) and urea compounds. This method has limited sensitivity, and is not suitable for early AKI detection [2],thus it is necessary to identify markers and methods adequate for the early detection of glomeruli, and/or tubule injury before the decreased glomerular filtration rate (GFR) is signalled by increased Crea concentration [3–6]. One such method is proteomic analysis, which compares the protein profile in normal urine with that typical for a given disease to select potential diagnostic, therapeutic, and prognostic biomarkers [7, 8]. With the decreased GFR and subsequent azotemia and uremia, AKI is among the most frequent complications of babesiosis in dogs, and may provide a natural model for identifying early and specific markers of kidney injury in this species [9–12]. Urinary proteins are a promising target for detecting kidney injury. Only a minimal amount of proteins is present in normal urine, due to the mechanical barrier of the glomerulus, and the reabsorption in the proximal tubules. Urinary total protein (UTP) contains proteins originating from filtered plasma, lower urinary tract, and kidney-derived proteins. High urinary protein concentration can be a result of nephron dysfunction, as healthy glomerular filtration barrier excludes proteins larger than 69 kDa, the molecular weight of albumin. Also it is worth noticing that positively charged proteins pass the glomelural barrier easier than the negatively charged ones. In conditions of disease, glomerular barrier gradually collapses, allowing large amounts of proteins of high, or intermediate weight pass into the ultrafiltrate. Proteins of small molecular weight (> 69 kDa) are freely filtered in the glomerulus, but are later reabsorbed by the kidney proximal tubules, therefore both primary and secondary tubular dysfunction can result in proteinuria [11].

Possible causes of acute kidney injury in dogs with babesiosis include anaemic hypoxia, hypovolemia, haemoglobinuric nephropathy, and myoglobinuric nephropathy secondary to rhabdomyolysis [13–15]. Anoxia, reduced renal blood flow, hypotension, and renal ischaemia probably play more important roles in the development of AKI than haemoglobinuria. Hypoxia results in greater renal tubular injury than haemoglobin, and the nephrotoxic effects of haemoglobin are highly individual [14]. Additionally, as shown by Zygner et al. [16], the increase in serum TNF-α concentration in dogs with canine babesiosis influences the development of hypotension and renal failure.

Moreover, as AKI naturally occurs during babesiosis in dogs, this situation could serve as a good model for select studies on AKI in humans. This hypothesis is supported by comparative analysis of urine proteomes in humans and dogs; many proteins related to human diseases, including kidney diseases, have been identified in canine urine [17–19]. In addition, domestic dogs (*Canis lupus familiaris*) are increasingly perceived as an excellent animal model for studying complex human diseases [20]. Canine DNA and protein sequences are much closer than mouse sequences to human sequences, suggesting that canine biology is more similar in many aspects to human biology than is mouse biology [21, 22]. It is also worth a mention that babesiosis is a zoonotic parasitic infection, and has similar clinical presentation to canine babesiosis [23].

This study aimed to identify proteins in the urine of dogs with subclinical kidney injury during the natural course of babesiosis, and to compare them with proteins in the control group to reveal potential biomarkers predicting renal injury before the presence of azotemia.

## Results

Based on the clinicopathological variables, all dogs with babesiosis met the criteria for early phase AKI [24]. They had proteinuria with UPC > 0.5, decreased urine specific gravity (average, 1.015) and significantly elevated uIgG/uCr, uTHP/uCr, and uRBP/uCr values, which indicated glomerular and tubular damage.

**Fig. 1:**
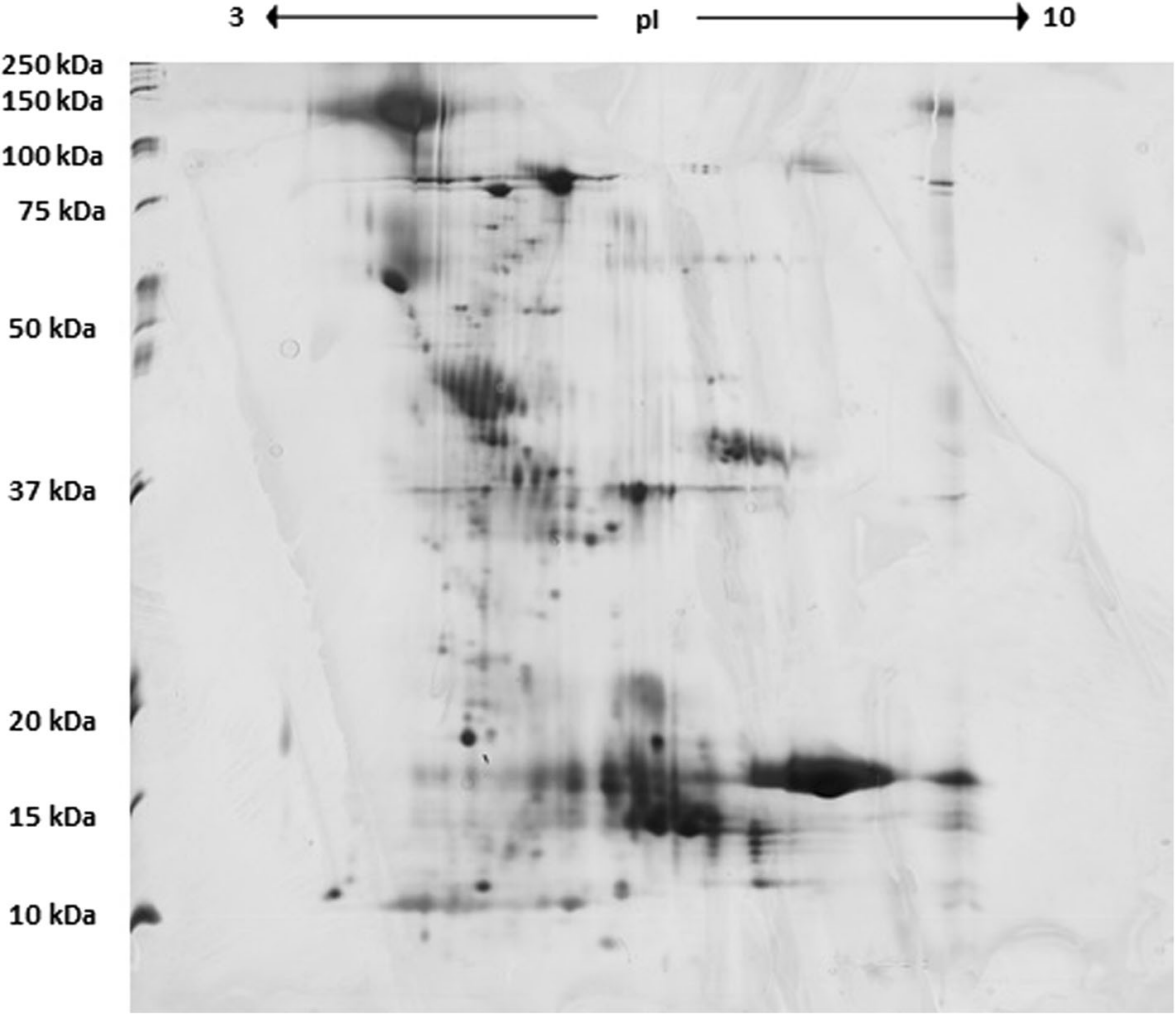
Image of 2DE gel from dogs with babesiosis

**Fig. 2:**
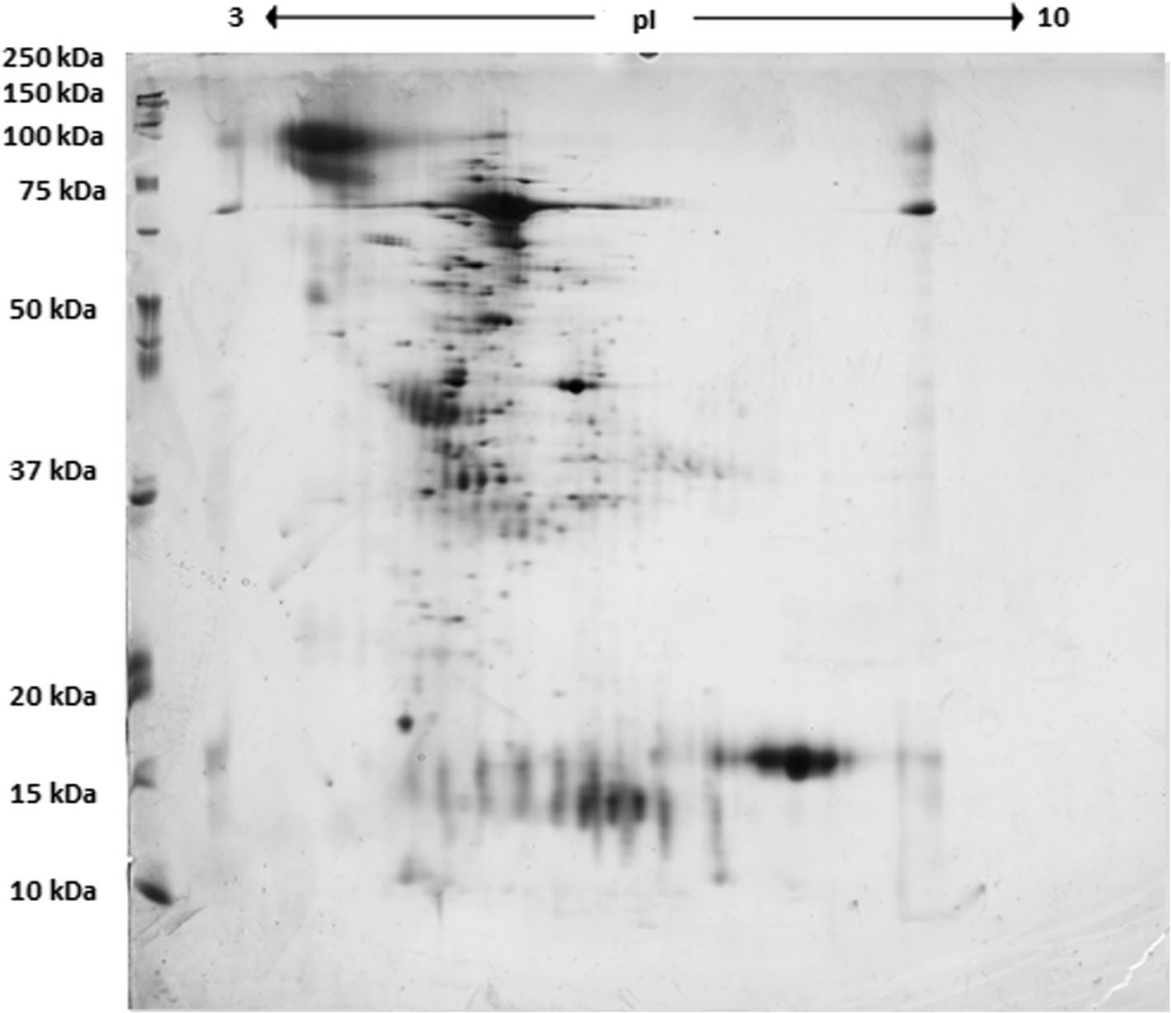
Image of 2DE gel from healthy dogs

In this study, 176 proteins were identified in pooled urine samples collected from healthy dogs, and 403 proteins were identified in pooled urine samples collected from dogs with babesiosis. Images of the gels obtained from dogs with babesiosis, and healthy dogs are shown in Figs. 1 and 2, respectively. Additional file 1: Tables S1 and Additional file 2: Table S2 contain lists of the proteins, along with their names, scores, molecular weights, number of matches, UniProt base accession numbers and hyperlinks (see Additional file 1: Table S1 and Additional file 2: Table S2). With Venn diagram software, which shows logical correlations between groups (http://bioinfogp.cnb.csic.es), 146 of the 176 proteins were assigned exclusively to healthy dogs, and 373 of the 403 proteins were exclusively assigned to dogs with babesiosis; 30 proteins were common for both groups*.* List of 30 common proteins between two groups has been presented in Supplementary data (see Additional file 3: Table S3). From 146 proteins found exclusively in healthy dogs, 128 were identified by Pantherdb software. According to molecular pathways analysis, those were listed in categories as follows: binding, catalytic activity, molecular function regulator, molecular transducer activity, sturctural molecule activity, transcription regulator activity, and transporter activity. Two most prominent molecular functions of those proteins were binding, and catalytic activity, consisting of 40 and 27 proteins, respectively.

To further evaluate the 373 proteins found in only the dogs with babesiosis, the Panther programme (http://www.pantherdb.org) was used to isolate 21 proteins from the *Canis familiaris* species, which were used to form a collection of potential diagnostic and pathophysiological biomarkers for this disease (Table 1). Further analysis of these 21 proteins led to the isolation of 8 proteins associated with 10 metabolic pathways, that were attributed to immune and inflammatory response development (Table 2). Further analysis indicated that a causal relationship network could be built by combining 5 of the 10 selected metabolic pathways and 4 of the 8 proteins with which the pathways were associated. These pathways included cadherins, gonadotropin releasing hormone receptors, inflammatory responses mediated by chemokine and cytokine signalling pathways, integrins, and TGF-β pathways and were linked by interleukin (IL)-13, bone morphogenetic protein 7, α2(1) collagen, and FER tyrosine kinase.

**Table 1:** List of *Canis familiaris* proteins identified in the urine of dogs with babesiosis by MALDI-TOF/TOF

Nr	Accession^a^	Protein name	GO molecular function
1	P06596	Phospholipase A2	phospholipase
2	A4Z944	Zinc finger BED domain-containing protein 5	transcription factor
3	O46392	Collagen alpha-2(I) chain	extracellular matrix structural constituent
4	Q9XSU4	40S ribosomal protein S11	structural constituent of ribosome
5	O97556	Rab GDP dissociation inhibitor beta	G-protein modulator acyltransferase
6	Q9TTY2	Tyrosine-protein kinase Fer	tyrosine kinese activity
7	P19006	Haptoglobin	hemoglobin binding
8	Q75V93	Calcitonin receptor-stimulating peptide 2	peptide hormone
9	Q2KNA0	Cytospin-A	structural component
10	P01321	Insulin	hormone
12	Q9N2I9	Mitochondrial uncoupling protein 3	oxidative phosphorylation
11	E2RK33	Glutamyl-tRNA (Gln) amidotransferase subunit C, mitochondrial	ligase
12	Q32KH5	N-acetylgalactosamine-6-sulfatase	hydrolase
13	P27597	T-cell surface glycoprotein CD3 epsilon chain	transmembrane signalling receptor
14	P24408	Ras-related protein Rab-9A	GTPase
15	P34819	Bone morphogenetic protein 7 (fragment)	growth factor
16	Q5TJE5	Ral guanine nucleotide dissociation stimulator-like 2	guanyl-nucleotide exchange factor
17	Q9N0W9	Interleukin-13	cytokine
18	Q8WMX5	Solute carrier family 15 member 1	transporter
19	O97578	Dipeptidyl peptidase 1 (fragment)	endopeptidase
20	Q861Y6	Nicolin-1	structural component
21	P17716	Islet amyloid polypeptide	hormone

^a^ in UniProt database (http://www.uniprot.org)

**Table 2:** List of metabolic pathways and associated urinary proteins in dogs with babesiosis

No.	Pathway	Protein
1.	CCKR signalling pathway	Calcitonin receptor-stimulating peptide 2
2.	Cadherin signalling pathway	Tyrosine-protein kinase Fer
3.	Gonadotropin-releasing hormone receptor pathway	Insulin/Bone morphogenetic protein 7 (fragment)
4.	Inflammatory response mediated by chemokines and cytokines	Interleukin 13
5.	Insulin/IGF pathway-mitogen activated protein kinase kinase/MAP kinase cascade	Insulin/Insulin-like growth factor
6.	Insulin/IGF pathway-protein kinase B signaling cascade	Insulin/Insulin-like growth factor
7.	Integrin signalling pathway	Collagen alpha-2(I) chain
8.	Ras pathway	Ral guanine nucleotide dissociation stimulator-like 2
9.	T cell activation	T cell surface glycoprotein CD3 epsilon chain
10.	TGF-beta signaling pathway	Bone morphogenetic protein 7 (fragment)

## Discussion

The final bioinformatic analysis of the urine proteome of dogs with babesiosis indicated that at least eight proteins (IL-13, bone morphogenetic protein 7, α2(1) collagen, tyrosine –protein kinase Fer, calcitonin receptor-stimulating peptide 2, insulin/insulin-like growth factor, ral guanine nucleotide dissociation stimulator-like 2 and T cell surface glycoprotein CD3 epsilon chain) are related to parasitic invasions and renal inflammatory responses. Non-specific immune responses are activated to limit the initial phase of parasitic invasion, or infection by pathogenic micro-organisms. Parasitic invasion initiates type Th2 immune response, characterised by the activation of Th2 lymphocytes, eosinophilia, basophilia, mast cells, and alternatively activated macrophages (AAM). This process is accompanied by the secretion of IgE antibodies and numerous cytokines, such as IL-3, IL-4, IL-5, IL-9, IL-10, IL-13 and TGF-β. It is well known that IL-13 plays a key role in regulating the anti-parasitic response [25] and is a primary factor that induces fibrosis in many chronic contagious and autoimmune diseases [26]. IL-13 increases the concentration of TGF-β, which leads to collagen deposition in lung and kidney tissues [27], by stimulating its production by macrophages via IL-13Rα2 [28, 29]. Fibrosis is considered the final stage in CKD development regardless of the primary cause, and the effector cells of this process include myofibroblasts generated from renal tubule epithelial cells by epithelial-mesenchymal transition (EMT) [30–33]. During this transition, cells lose polarity upon losing certain communication abilities, and degrading the basement membrane. Adhesive molecules that bind both epithelial cells and the basement membrane, such as E-cadherin and integrins, are replaced by mesenchymal cell markers, such as N-cadherin, nonstriated muscle α-actin, vimentin, fibronectin and collagen I. In an early inflammatory environment, EMT maintains renal tissue homoeostasis by inducing structural regeneration and reconstruction after harmful stress. Long-term support of EMT leads to fibrous degeneration as well as structural and functional tissue and organ disorders [34, 35]. Pleiotropic TGF-β molecules and BMPs belonging to the transforming growth factor-β superfamily (TGF-βSF) participate in one of the most well-known signalling pathways in EMT [36–38]. Increased TGF-β levels lead to loss of the epithelial phenotype, acquisition of the mesenchymal phenotype and collagen accumulation. Serine-threonine kinase receptors and cytoplasmic proteins (Smads) participate in transmitting TGF-β/BMP pathway signals. Smad3, which is induced by TGF-β stimulation, can bind the Col1A2 gene promoter to activate the expression of type 1α2 collagen, which may accumulate in interstitial tissue and contribute to extracellular matrix (ECM) accumulation, leading to fibrous degeneration of the organ [39]. On the other hand, BMP-7 inhibits fibrosis, exerts anti-inflammatory effects and stimulates the regeneration of damaged kidney tissues [40]. In experimental systems, BMP-7 recombinant protein expression or overexpression inhibits fibrosis in diabetic nephropathy or AKI, TGF-β-initiated EMT and E-cadherin suppression [41]. BMP-7 exerts an anti-inflammatory effect by inhibiting neutrophil, monocyte and macrophage infiltration and activity, as well as by repressing the expression of the proinflammatory cytokines IL-6 and IL-1β, and the proinflammatory chemokines MCP1 and IL-8 [41].

The epsilon chain is one of the four subunits of the CD3 protein complex that combines participation in the activation of T cells after antigen binding. The CD3 epsilon protein is also expressed in proximal and distal tubules, and Henle loops. The presence of this protein in renal tubules results from the participation in the active transport of sodium or hydrogen ions as sodium or proton pumps [42]. It is also believed that CD3 epsilon protein may be involved in communication of signal transduction, similar to T lymphocytes.

Insulin-like growth factor-1 (IGF-1) is produced by the collecting duct of the adult kidney. Its receptors are present in glomeruli, and on the basolateral membrane of renal proximal tubular cells. IGF-1 promotes cell proliferation and inhibits apoptosis by activation of either phosphatidylinositol-3 kinase (PI3K/Akt), or extracellular signal-regulated kinase (ERK)/mitogen-Activated Protein Kinase (MAPK) pathway [43]. Renal IGF-1 has been shown to decrease in course of ischemic injury, and administration of its exogenous form has been reported to accelerate recovery from ischemic acute renal failure. Reduced apoptosis, and enhanced proliferation of tubular epithelial cells were the proposed mechanisms of action responsible for this beneficial effect [44]. The presence of insulin in association with the IGF-I pathway demonstrated in our studies may indicate with some probability, that IGFBP-7 could be a potential biomarker of acute kidney injury in dogs with babesiosis. However, the exact mechanisms underlying this process are not completely understood.

Haptoglobin, and acute phase protein, also appeard among 21 proteins identified in the urine of dogs with babesiosis. Although it was not included in the final network of close connections selected by the Panther system, haptoglobin is considered to be one of the valuable markers used in the assessment of the course of babesiosis in dogs. In the other study, dogs with babesisois had reduced serum level of haptoglobin in relation to normal values ​​ [45, 46]. In this context, it is worth to ask a question about the correlation between a decreased serum level of haptoglobin and its level in excreted urine.

In this study, protein analysis was performed by mass spectrometry without any pre-treatment of urine. Looking at the gene ontology map derived from the dataset, it appears that urine proteome in dogs with babesiosisis composed of several clusters of proteins. Although our study has a major limitation of using a pooled samples, we beleive that our findings complete in some way the description of urinary signs of a clinical conditions of renal tissue in dogs with babsiosis. Verifying their significance in the diagnosis and prognosis of the disease requires further study.

## Conclusions

In summary, to the best of our knowledge, this study is the first to comprehensively analyse the urinary proteome of dogs with babesiosis, demonstrating the association of the identified proteins with this disease, and kidney injury. Urine interleukin-13, bone morphogenetic protein 7, α2(1) collagen and tyrosine-protein kinase Fer are potential biomarkers of kidney injury during babesiosis in dogs that might indicate early renal injury; however, further studies are needed to verify their significance in the diagnosis and prognosis of the disease. Functional analysis of these four proteins indicates that epithelial-mesenchymal transition (EMT) might play an important role in the mechanisms underlying pathological changes in renal tissues during the course of babesiosis.

## Methods

### Animals and sample collection

Dogs were enrolled during routine admission to Faculty of Veterinary Medicine clinics at the University of Life Sciences in Lublin. Informed consent was obtained from the owners prior to clinical investigations and sample collection. The studies were reviewed and approved by the Ethics Committee of the University of Life Sciences in Lublin (Poland) No 70/2018. All relevant data for inclusion criteria for dogs and values of urine parameters and urinary biomarkers used in the study have already been published [47]. Briefly, the study involved 20 mixed-breed dogs (10 males, 10 females) weighing 5–8 kg (median, 6.2 kg) and aged 2–7 years (median, 4.35 years) that were divided into two groups. All dogs underwent individual clinical and laboratory tests to determine their health status, and to identify signs of kidney damage, particularly in the diseased group. Group 1 (study group, *n* = 10; five males and five females) consisted of dogs naturally infected with *B. canis*, while group 2 (control group, *n* = 10; five males and five females) consisted of healthy dogs [47]. All dogs in group 1 showed symptoms of babesiosis (apathy, anorexia, changes in urine colour, and pale mucous membranes), and haematology analysis revealed thrombocytopenia (platelets 12–88 × 10^9^/l) and anaemia (erythrocytes 3.5–5.3 × 10^12^/l.) All dogs were nonazotemic, and the serum creatinine concentration remained within the reference range. All dogs in this group had *Babesia*-positive blood smears, and infection was additionally confirmed by PCR according to the protocol described in other studie [12, 16]. Possible co-infections (borreliosis, anaplasmosis, ehrlichiosis) were excluded in all dogs based on PCR and ELISA results [48]. All dogs in group 1 were successfully treated with imidocarb (5 mg/kg s.c.). Dogs in group 2 were clinically healthy and were referred to the clinic for vaccination purposes. Blood smear analysis and PCR for *B. canis* gave negative results for all animals in group 2. Voided midstream urine samples were collected in the morning before the treatment with imidocarb, and each sample was centrifuged on the day of collection at 500×g for 10 min at 4 °C. The supernatants were removed, and protease inhibitors were added (Protease Inhibitor Cocktail, Roche Diagnostic Corp.). Urine protein and Cr concentration were measured by the enzymatic colorimetric method (BS-130 analyser, Mindray), and basic urinalysis with microscopic sediment analysis was performed on fresh urine samples. Urine specific gravity (USG) was measured using a refractometer. The remaining urine was frozen at − 80 °C for further analysis. Macroscopic evaluation of urine in group 1 showed yellow to dark brown samples, while all group 2 samples were yellow. Urine protein analysis revealed proteinuria in eight of the 10 group 1 dogs, and eight dogs in this group also had a urine protein/creatinine ratio > 0.5. Urine dipstick analysis showed haemoglobinuria in seven of the 10 group 1 dogs, which was severe (+++) in two dogs. Urine specific gravity was decreased in all diseased dogs, with an average value of 1.015 (Table 3). No dogs in the control group had proteinuria or haemoglobinuria. Statistically higher concentrations of urinary biomarkers (uIgG/uCr, uTHP/uCr, and uRBP/uCr) were found in the urine samples from all dogs with babesiosis compared to those from the control animals (*p* < 0.05), indicating dysfunctional glomerular and tubular kidney regions (Table 3). For proteomic analysis, 10 individual urine samples (0.5 ml each) from groups 1 (affected dogs) and 2 (healthy dogs) were collected and pooled. Pooled sample was made by mixing the same amount of protein of each tear fluid sample. Each pooled urine sample was subjected to desalting on the filter to enable quick ultrafiltration with a high-density coefficient (Amicon Ultra Merck). Protein concentrations were measured with a microlitre spectrophotometer (NANO), and the urine samples were then prepared and subjected to 2D electrophoresis. Each individual gel spot was then analysed by mass spectrometry with the MALDI-TOF (matrix-assisted laser desorption ionization–time of flight) technique.

**Table 3:** Urinary parameters of renal function and concentration of urinary markers in dogs with babesiosis (group 1) and healthy dogs (group 2) (expressed as the median and range)

Variable	Group 1 (*n* = 10)	Group 2 (*n* = 10)	*P* value
uCr [mg/dl]	58.68 (3.24–120.58)	135.64 (53.91–212.82)	0.02
Specific gravity	1.015 (1.010–1.030)	1.030 (1.015–1.045)	0.02
UPC [mg/mg]	2.3 (0.48–6.24)	0.2 (0.05–0.40)	0.03
uRBP/uCr [mg/g]	24.65 (0.06–76.21)	0.2 (0.09–0.3)	0.03
uTHP/uCr [mg/g]	1.50 (0.13–7.80)	0.2 (0.09–0.3)	0.03
uIgG/uCr [mg/g]	120.78 (0–394.31)	0	0.02

*UPC* urine protein-to-creatinine ratio, *uRBP* urinary retinol binding protein, *uTHP* urinary Tamm-Horsfal protein, *uIgG* urinary immunoglobulin G, *uCrea* urinary creatinine

### 2D electrophoresis

Two-dimensional electrophoresis was used to separate the proteins in the tested urine samples [49]. Preliminary tests showed that the optimum amount of protein for 2D electrophoresis is 85 μg; thus, this amount of protein was broken down via a precipitation and purification kit (ReadyPrep™ 2-D Cleanup Kit, Bio-Rad, Warsaw, Poland). The obtained protein pellets were then dissolved in rehydration buffer, and the resulting solutions were applied to a rehydration plate and covered with 17-cm immobilized pH gradient (IPG) linear strips for isoelectric focusing (pH 3–10, Bio-Rad). The gel on the strips was soaked with the protein sample, and the strips were removed after a 12-h rehydration period and then subjected to electrophoresis in the first dimension (IEF-100 Hoefer; 250 V/30 min; 10,000 V/3 h; 60 kV/hr., with a current limit of 50 μA/strip hr). Under the influence of the electric field, proteins in the strips migrated to the location corresponding to their isoelectric point. After separation, the IPG strips were prepared for electrophoresis in the second dimension to separate the proteins by molecular mass. Vertical electrophoretic separation was performed in 12.5% polyacrylamide gels with the following current parameters: 600 V/30 mA/100 W in an electrophoretic chamber (PROTEAN® II xi, Bio-Rad). The obtained gels were subjected to a standard colouring procedure with silver in the presence of formaldehyde as a regulator. The protein spots were cut out of the gels, decolourized, reduced and alkylated using dithiothreitol and iodoacetamide [50]. Gel pieces containing proteins were subjected to digestion to obtain shorter peptide fragments. Trypsin digestion occurred in 50 mM ammonium bicarbonate buffer at 37 °C for 12 h (Promega, Trypsin Gold, Mass Spectrometry Grade, Technical Bulletin) [51]. The obtained peptides were subsequently eluted from the gel with a water/acetonitrile/TFA solution (v:v 450:500:50). The extracted peptides were purified using C18 Zip-TIP pipette tips according to the manufacturer’s instructions (Merck Chemicals, Billerica, MA, USA, PR 02358, Technical Note) and applied to the MTP AnchorChip 384 plate (Bruker, Bremen, Germany).

### Mass spectrometry

After the protein samples were dried on the MTP AnchorChip 384 plate, the surface was covered with a super-saturated solution of α-cyano-4-hydroxycinnamic acid (HCCA, Bruker), functioning as a matrix mediating energy transmission to the sample. Simultaneously, 0.5 μl of a peptide standard was applied to the calibration fields (Peptide Calibration Standard II, Bruker), which were also covered with the matrix solution. Spectrometric analysis was performed using an UltrafleXtreme III MALDI-TOF/TOF (Bruker), and flexControl 3.3 (Bruker) software was applied for mass spectra collection. The obtained peptides were subjected to mild ionization using the MALDI-TOF instrument in linear mode within the 900–4000 Da mass scope in reflectron mode. The obtained mass spectra were analysed with flexAnalysis 3.4 (Bruker) software as follows: smoothing (Savitsky-Golay method), baseline subtraction (Top Hat baseline algorithm), and peak geometry (Stanford Network Analysis Platform (SNAP) algorithm). All peaks with a signal to noise ratio > 3 qualified for further analysis. Experimental data were analysed using the abovementioned software to exclude peaks originating from trypsin or environmental pollution. To ensure correct identification, the selection of possible post-translational modifications using BioTools 3.2 (Bruker) was essential. Post-translational modifications are derived from both the methodology and metabolic processes in the patients. The obtained spectra were compared to the Swiss-Prot database restricted to “bony vertebrate” taxa using Mascot 2.2 software with a maximum error of 0.3 Da. The results with Mascot scores above 62 were considered statistically significant (*p* ≤ 0.05). If this threshold was not reached, the fragment ion spectra of chosen peptides were subjected to fragmentation in tandem spectrometry mode [52, 53].

### Bioinformatic analysis

Venn diagrams were used to show differences between gene lists of healthy and diseased dogs where the UniProt accession numbers were use d [54].

By means of this software it was possible to obtain subset of proteins assigned exclusively to healthy dogs, subset of proteins common to both groups and subset of proteins assigned exlusively to dogs with babesiosis.

Then to study the biological pathway networks and functional classification the UniProt accession numbers of the protein subset assigned to dogs with babesiosis were entered into Panther Classification System [55] . Analysis was carried out selecting *Canis lupus familiaris* database.

## Supplementary information

**Additional file 1 Table S1.** Proteins identified in urine from healthy dogs.

**Additional file 2 Table S2.** Proteins identified in urine from dogs with babesiosis.

**Additional file 3 Table S3.** Common proteins for babesiosis and healthy dogs.

## Data Availability

The datasets used and analysed during the current study are available from the corresponding author on reasonable request.

## Abbreviations

2D: Two-dimensional
AKI: Acute kidney injury
CD3: Cluster of differentiation 3,
CKD: Chronic kidney disease
Cr: Creatinine
ECM: Extracellular matrix
EMT: Epithelial-mesesnchymal transition
GFR: Glomerular filtration rate
IGF-I: Insulin-like growth factor
Il13: Interleukin 13
MALDI-TOF: Matrix-assisted laser desorption ionization time of flight
TGF-β: Transforming growth factor
uIgG: Urinary immunoglobulin G
UPC: Urine protein to creatinine ratio
uRBP: Urinary retinol-binding protein
USG: Urine specific gravity
uTHP: Urinary TammHorsfall Protein
