# Supplementary material for: Urinary proteome of dogs with kidney injury during babesiosis
Source: BMC Vet Res. 2019 Dec 4;15:439. doi: 10.1186/s12917-019-2194-0 (PMC6894246; doi:10.1186/s12917-019-2194-0)
Supplement: Supplementary file 1 — Additional file 1 Table S1. Proteins identified in urine from healthy dogs. [file 12917_2019_2194_MOESM1_ESM.docx]

| Table S1. Proteins identified in urine of healthy dogs. | | | | | |
| --- | --- | --- | --- | --- | --- |
| Protein name | Score | Mass | Matches | Access no. | Hyperlink |
| Endophilin-A2 | **64** | 41.7 | 9 | **Q2KJA1** | http://www.uniprot.org/uniprot/Q2KJA1 |
| BTB/POZ domain-containing protein KCTD1 | **66** | 29.7 | 7 | **Q719H9** | http://www.uniprot.org/uniprot/Q719H9 |
| Prolyl 3-hydroxylase 3 | **52** | 82.6 | 11 | **Q8IVL6** | http://www.uniprot.org/uniprot/Q8IVL6 |
| Essential MCU regulator | **54** | 11.5 | 4 | **Q2M2S2** | http://www.uniprot.org/uniprot/Q2M2S2 |
| C-X-C motif chemokine 3 | **43** | 11.3 | 4 | **Q10746** | http://www.uniprot.org/uniprot/Q10746 |
| Desmin | **65** | 53.3 | 10 | **Q5XFN2** | http://www.uniprot.org/uniprot/Q5XFN2 |
| Uromodulin | **65** | 72.9 | 13 | **Q862Z3** | http://www.uniprot.org/uniprot/Q862Z3 |
| Heat shock factor-binding protein 1 | **51** | 8.5 | 4 | **O75506** | http://www.uniprot.org/uniprot/O75506 |
| Phosphoglucomutase-2 | **45** | 69.9 | 11 | **Q7TSV4** | http://www.uniprot.org/uniprot/Q7TSV4 |
| Methylmalonyl-CoA mutase, mitochondrial | **50** | 83.6 | 12 | **Q9GK13** | http://www.uniprot.org/uniprot/Q9GK13 |
| Histone H1t | **51** | 22.1 | 6 | **P40286** | http://www.uniprot.org/uniprot/P40286 |
| General transcription factor II-I | **50** | 110.6 | 8 | **A7MB80** | http://www.uniprot.org/uniprot/A7MB80 |
| Zinc finger protein 106 | **68** | 210.8 | 19 | **O88466** | http://www.uniprot.org/uniprot/O88466 |
| Protein CutA | **76** | 19.2 | 6 | **O60888** | http://www.uniprot.org/uniprot/O60888 |
| Protein Lines homolog 1 | **58** | 87.5 | 11 | **Q8NG48** | http://www.uniprot.org/uniprot/Q8NG48 |
| Dihydropyrimidinase-related protein 1 | **48** | 62.5 | 9 | **Q14194** | http://www.uniprot.org/uniprot/Q14194 |
| Interleukin-22 | **43** | 20.3 | 4 | **Q9GZX6** | http://www.uniprot.org/uniprot/Q9GZX6 |
| BTB/POZ domain-containing protein KCTD1 | **46** | 29.7 | 4 | **Q719H9** | http://www.uniprot.org/uniprot/Q719H9 |
| Ribosome-binding protein 1 | **48** | 164.8 | 9 | **Q28298** | http://www.uniprot.org/uniprot/Q28298 |
| Glycogen debranching enzyme | **43** | 176.9 | 12 | **Q2PQH8** | http://www.uniprot.org/uniprot/Q2PQH8 |
| 6-phosphofructo-2-kinase/fructose-2,6-bisphosphatase 3 | **67** | 54.2 | 9 | **Q28901** | http://www.uniprot.org/uniprot/Q28901 |
| 60S ribosomal protein L37 | **58** | 11.3 | 8 | **P79244** | http://www.uniprot.org/uniprot/P79244 |
| Interleukin-11 | **46** | 21.6 | 6 | **P47873** | http://www.uniprot.org/uniprot/P47873 |
| Vascular cell adhesion protein 1 | **63** | 82.3 | 13 | **P19320** | http://www.uniprot.org/uniprot/P19320 |
| Ig heavy chain V region AC38 205.12 | **66** | 13 | 4 | **P06330** | http://www.uniprot.org/uniprot/P06330 |
| Collagen alpha-1(XXV) chain | **88** | 65.1 | 13 | **Q9BXS0** | http://www.uniprot.org/uniprot/Q9BXS0 |
| Sphingosine 1-phosphate receptor 3 | **51** | 43 | 8 | **Q99500** | http://www.uniprot.org/uniprot/Q99500 |
| Vascular cell adhesion protein 1 | **51** | 82.3 | 11 | **P19320** | http://www.uniprot.org/uniprot/P19320 |
| Vacuolar protein sorting-associated protein 4B | **41** | 49.6 | 6 | **P46467** | http://www.uniprot.org/uniprot/P46467 |
| SPRY domain-containing protein 7 | **51** | 22.2 | 5 | **Q2T9X3** | http://www.uniprot.org/uniprot/Q2T9X3 |
| Myoglobin | **48** | 17.3 | 7 | **P02185** | http://www.uniprot.org/uniprot/P02185 |
| Retinol-binding protein 2 | **43** | 15.8 | 5 | **Q08652** | http://www.uniprot.org/uniprot/Q08652 |
| Mesenteric estrogen-dependent adipogenesis protein | **53** | 34.6 | 6 | **A4IFN2** | http://www.uniprot.org/uniprot/A4IFN2 |
| NADH dehydrogenase [ubiquinone] 1 alpha subcomplex subunit 10, mitochondrial | **49** | 40.9 | 6 | **Q0MQB6** | http://www.uniprot.org/uniprot/Q0MQB6 |
| Nucleoside diphosphate kinase A | **51** | 17.3 | 5 | **Q05982** | http://www.uniprot.org/uniprot/Q05982 |
| PR domain zinc finger protein 12 | **49** | 40.7 | 6 | **A2AJ77** | http://www.uniprot.org/uniprot/A2AJ77 |
| Gastric inhibitory polypeptide receptor | **53** | 54 | 8 | **P48546** | http://www.uniprot.org/uniprot/P48546 |
| Carbohydrate sulfotransferase 1 | **54** | 47.5 | 9 | **Q9EQC0** | http://www.uniprot.org/uniprot/Q9EQC0 |
| Ras-specific guanine nucleotide-releasing factor 1 | **50** | 146.3 | 13 | **Q13972** | http://www.uniprot.org/uniprot/Q13972 |
| Coiled-coil domain-containing protein 184 | **39** | 20.7 | 3 | **Q52MB2** | http://www.uniprot.org/uniprot/Q52MB2 |
| NADH dehydrogenase [ubiquinone] 1 alpha subcomplex subunit 4-like 2 | **42** | 10.1 | 3 | **Q9NRX3** | http://www.uniprot.org/uniprot/Q9NRX3 |
| Elongation factor 1-beta | **59** | 25 | 7 | **Q5E983** | http://www.uniprot.org/uniprot/Q5E983 |
| Uncharacterized protein C12orf60 homolog | **52** | 28.3 | 10 | **Q810N5** | http://www.uniprot.org/uniprot/Q810N5 |
| Serum albumin | **44** | 70.6 | 10 | **P49822** | http://www.uniprot.org/uniprot/P49822 |
| DNA-binding protein RFX5 | **52** | 65.7 | 11 | **P48382** | http://www.uniprot.org/uniprot/P48382 |
| C2 domain-containing protein 3 | **50** | 262.6 | 15 | **Q4AC94** | http://www.uniprot.org/uniprot/Q4AC94 |
| Protein deglycase DJ-1 | **55** | 20.1 | 6 | **Q95LI9** | http://www.uniprot.org/uniprot/Q95LI9 |
| Calmodulin-regulated spectrin-associated protein 1 | **54** | 179.9 | 12 | **D3Z8E6** | http://www.uniprot.org/uniprot/D3Z8E6 |
| Zinc finger protein 101 | **49** | 51.9 | 7 | **Q8IZC7** | http://www.uniprot.org/uniprot/Q8IZC7 |
| Mini-chromosome maintenance complex-binding protein | **51** | 73.8 | 7 | **Q9BTE3** | http://www.uniprot.org/uniprot/Q9BTE3 |
| Protein SOX-16 (Fragment) | **46** | 6.9 | 5 | **Q62247** | http://www.uniprot.org/uniprot/Q62247 |
| Essential MCU regulator, mitochondrial | **45** | 11.5 | 4 | **Q2M2S2** | http://www.uniprot.org/uniprot/Q2M2S2 |
| G protein-coupled receptor kinase 7 | **52** | 62.6 | 7 | **Q9Z2G7** | http://www.uniprot.org/uniprot/Q9Z2G7 |
| Golgi SNAP receptor complex member 1 | **49** | 28.6 | 11 | **Q2TBU3** | http://www.uniprot.org/uniprot/Q2TBU3 |
| Metaxin-2 | **52** | 30.1 | 5 | **O88441** | http://www.uniprot.org/uniprot/O88441 |
| Glutathione S-transferase Mu 1 | **52** | 26.1 | 7 | **P10649** | http://www.uniprot.org/uniprot/P10649 |
| Calpain-2 catalytic subunit | **58** | 80.7 | 9 | **Q27971** | http://www.uniprot.org/uniprot/Q27971 |
| Pleckstrin homology domain-containing family G member 4B | **56** | 141.6 | 15 | **Q96PX9** | http://www.uniprot.org/uniprot/Q96PX9 |
| Profilin-4 | **52** | 14.6 | 6 | **Q9D6I3** | http://www.uniprot.org/uniprot/Q9D6I3 |
| EH domain-containing protein 4 | **52** | 61.4 | 12 | **Q9H223** | http://www.uniprot.org/uniprot/Q9H223 |
| Hepcidin | **59** | 9.3 | 5 | **Q8MJ80** | http://www.uniprot.org/uniprot/Q8MJ80 |
| Glycine receptor subunit beta | **52** | 56.8 | 7 | **P48167** | http://www.uniprot.org/uniprot/P48167 |
| Sulfotransferase 4A1 | **51** | 33.4 | 8 | **P63046** | http://www.uniprot.org/uniprot/P63046 |
| Unconventional myosin-Id | **59** | 116.9 | 10 | **O94832** | http://www.uniprot.org/uniprot/O94832 |
| Actin-related protein 2/3 complex subunit 3 | **48** | 20.8 | 6 | **Q3T035** | http://www.uniprot.org/uniprot/Q3T035 |
| Actin-related protein 2/3 complex subunit 3 | **48** | 20.8 | 6 | **Q3T035** | http://www.uniprot.org/uniprot/Q3T035 |
| Autophagy-related protein 16-1 | **49** | 68.9 | 7 | **Q676U5** | http://www.uniprot.org/uniprot/Q676U5 |
| Carbonic anhydrase 5B, mitochondrial | **61** | 36.8 | 5 | **Q9Y2D0** | http://www.uniprot.org/uniprot/Q9Y2D0 |
| Putative olfactory receptor 2B3 | **50** | 36.2 | 4 | **O76000** | http://www.uniprot.org/uniprot/O76000 |
| Zinc finger protein 75D | **51** | 60.2 | 10 | **P51815** | http://www.uniprot.org/uniprot/P51815 |
| Trafficking protein particle complex subunit 1 | **57** | 16.9 | 6 | **Q17QI1** | http://www.uniprot.org/uniprot/Q17QI1 |
| Golgi SNAP receptor complex member 1 | **52** | 28.6 | 9 | **Q2TBU3** | http://www.uniprot.org/uniprot/Q2TBU3 |
| Zinc finger protein 491 | **50** | 52.9 | 9 | **Q8N8L2** | http://www.uniprot.org/uniprot/Q8N8L2 |
| Cytoskeleton-associated protein 2-like | **61** | 83.4 | 12 | **A5PK21** | http://www.uniprot.org/uniprot/A5PK21 |
| Retinoic acid receptor RXR-beta (Fragment) | **52** | 49.8 | 9 | **P49743** | http://www.uniprot.org/uniprot/P49743 |
| Apolipoprotein A-II | **54** | 11.3 | 4 | **E2RAK7** | http://www.uniprot.org/uniprot/E2RAK7 |
| Tubulin polymerization-promoting protein family member 2 | **50** | 18.5 | 6 | **Q4R3A0** | http://www.uniprot.org/uniprot/Q4R3A0 |
| Bcl-2-like protein 2 | **49** | 20.9 | 6 | **Q1RMX3** | http://www.uniprot.org/uniprot/Q1RMX3 |
| Mini-chromosome maintenance complex-binding protein | **52** | 73.8 | 16 | **A5PJM5** | http://www.uniprot.org/uniprot/A5PJM5 |
| Alpha-2,8-sialyltransferase 8F | **55** | 45.4 | 8 | **P61647** | http://www.uniprot.org/uniprot/P61647 |
| H-2 class I histocompatibility antigen, K-B alpha chain | **62** | 41.7 | 11 | **P01901** | http://www.uniprot.org/uniprot/P01901 |
| Fibroleukin | **53** | 50.8 | 9 | **Q14314** | http://www.uniprot.org/uniprot/Q14314 |
| Phosphatidylethanolamine-binding protein 2 | **52** | 21.7 | 6 | **Q8VIN1** | http://www.uniprot.org/uniprot/Q8VIN1 |
| Zinc finger and SCAN domain-containing protein 5A | **63** | 56.9 | 8 | **Q9BUG6** | http://www.uniprot.org/uniprot/Q9BUG6 |
| Fructose-1,6-bisphosphatase 1 | **54** | 37 | 5 | **Q3SZB7** | http://www.uniprot.org/uniprot/Q3SZB7 |
| Beta-defensin 107A | **51** | 7.9 | 3 | **A4H217** | http://www.uniprot.org/uniprot/A4H217 |
| Golgi SNAP receptor complex member 1 | **62** | 28.6 | 10 | **Q62931** | http://www.uniprot.org/uniprot/Q62931 |
| Zinc finger protein 624 | **63** | 102.5 | 13 | **Q9P2J8** | http://www.uniprot.org/uniprot/Q9P2J8 |
| Prelamin-A/C | **62** | 74.6 | 12 | **P48679** | http://www.uniprot.org/uniprot/P48679 |
| Aspartate-tRNA ligase, cytoplasmic | **51** | 57.5 | 18 | **P15178** | http://www.uniprot.org/uniprot/P15178 |
| Beta-lactoglobulin | **69** | 20.6 | 6 | **Q29146** | http://www.uniprot.org/uniprot/Q29146 |
| ATP synthase subunit alpha, mitochondrial | **63** | 59.8 | 16 | **P25705** | http://www.uniprot.org/uniprot/P25705 |
| RUN and FYVE domain-containing protein 2 | **55** | 70.8 | 18 | **Q8R4C2** | http://www.uniprot.org/uniprot/Q8R4C2 |
| Pyridine nucleotide-disulfide oxidoreductase domain-containing protein 2 | **57** | 63.5 | 10 | **Q3U4I7** | http://www.uniprot.org/uniprot/Q3U4I7 |
| Profilin-3 | **51** | 15 | 7 | **Q8R4C2** | http://www.uniprot.org/uniprot/Q9DAD6 |
| Prolyl 3-hydroxylase 3 | **62** | 82.6 | 10 | **Q8IVL6** | http://www.uniprot.org/uniprot/Q8IVL6 |
| Tumor susceptibility gene 101 protein | **56** | 44.1 | 7 | **Q99816** | http://www.uniprot.org/uniprot/Q99816 |
| Vascular cell adhesion protein 1 | **51** | 82.4 | 10 | **P29533** | http://www.uniprot.org/uniprot/P29533 |
| Ataxin-7 | **53** | 93.8 | 8 | **Q8R4I1** | http://www.uniprot.org/uniprot/Q8R4I1 |
| Gamma-aminobutyric acid receptor subunit alpha-1 | **53** | 52.1 | 6 | **P08219** | http://www.uniprot.org/uniprot/P08219 |
| 43 kDa receptor-associated protein of the synapse | **63** | 47.6 | 10 | **P12672** | http://www.uniprot.org/uniprot/P12672 |
| Protein-arginine deiminase type-2 | **57** | 76 | 9 | **P20717** | http://www.uniprot.org/uniprot/P20717 |
| Heat shock factor-binding protein 1 | **53** | 8.6 | 6 | **Q9CQZ1** | http://www.uniprot.org/uniprot/Q9CQZ1 |
| Non-homologous end-joining factor 1 | **55** | 34.1 | 7 | **Q6AYI4** | http://www.uniprot.org/uniprot/Q6AYI4 |
| Microtubule-associated protein RP/EB family member 1 | **51** | 30.1 | 10 | **Q5R7Z5** | http://www.uniprot.org/uniprot/Q5R7Z5 |
| Protein kish-A | **56** | 8.4 | 6 | **Q9CR64** | http://www.uniprot.org/uniprot/Q9CR64 |
| Ubiquitin carboxyl-terminal hydrolase 14 | **56** | 56.3 | 9 | **P40826** | http://www.uniprot.org/uniprot/P40826 |
| Cap-specific mRNA (nucleoside-2'-O-)-methyltransferase 1 | **64** | 96.6 | 12 | **Q9DBC3** | http://www.uniprot.org/uniprot/Q9DBC3 |
| DNA dC->dU-editing enzyme APOBEC-3G | **65** | 45.9 | 8 | **Q694B9** | http://www.uniprot.org/uniprot/Q694B9 |
| Annexin A10 | **63** | 37.8 | 8 | **Q9UJ72** | http://www.uniprot.org/uniprot/Q9UJ72 |
| Cysteine and glycine-rich protein 2 | **57** | 21.8 | 6 | **P97314** | http://www.uniprot.org/uniprot/P97314 |
| Calmodulin-regulated spectrin-associated protein 1 | **50** | 179.9 | 15 | **D3Z8E6** | http://www.uniprot.org/uniprot/D3Z8E6 |
| Tyrosine-protein phosphatase non-receptor type 12 | **58** | 87.2 | 7 | **P35831** | http://www.uniprot.org/uniprot/P35831 |
| Dual specificity phosphatase DUPD1 | **55** | 24.3 | 6 | **P0C595** | http://www.uniprot.org/uniprot/P0C595 |
| Protein cereblon | **53** | 50.1 | 10 | **Q5R6Y2** | http://www.uniprot.org/uniprot/Q5R6Y2 |
| Testis-expressed sequence 33 protein | **53** | 30.8 | 5 | **O43247** | http://www.uniprot.org/uniprot/O43247 |
| Complexin-3 | **51** | 17.6 | 7 | **Q8WVH0** | http://www.uniprot.org/uniprot/Q8WVH0 |
| Plasmalemma vesicle-associated protein | **61** | 50.6 | 12 | **Q9WV78** | http://www.uniprot.org/uniprot/Q9WV78 |
| Calcium/calmodulin-dependent protein kinase II inhibitor 1 | **53** | 8.6 | 4 | **A7MBG3** | http://www.uniprot.org/uniprot/A7MBG3 |
| BTB/POZ domain-containing protein KCTD1 | **56** | 29.7 | 6 | **Q719H9** | http://www.uniprot.org/uniprot/Q719H9 |
| Threonine synthase-like 2 | **62** | 54.8 | 6 | **Q86YJ6** | http://www.uniprot.org/uniprot/Q86YJ6 |
| Probable tRNA pseudouridine synthase 1 | **65** | 36.6 | 7 | **Q5M934** | http://www.uniprot.org/uniprot/Q5M934 |
| Ras-related protein Rab-7a | **51** | 23.8 | 8 | **P51149** | http://www.uniprot.org/uniprot/P51149 |
| Essential MCU regulator, mitochondrial | **52** | 11.5 | 4 | **Q2M2S2** | http://www.uniprot.org/uniprot/Q2M2S2 |
| Golgi SNAP receptor complex member 1 | **51** | 28.6 | 5 | **O88630** | http://www.uniprot.org/uniprot/O88630 |
| Dual specificity phosphatase DUPD1 | **54** | 25.5 | 8 | **Q68J44** | http://www.uniprot.org/uniprot/Q68J44 |
| GTP-binding protein Rheb | **53** | 20.5 | 5 | **Q921J2** | http://www.uniprot.org/uniprot/Q921J2 |
| Radical S-adenosyl methionine domain-containing protein 2 | **60** | 42.4 | 10 | **Q9MZU4** | http://www.uniprot.org/uniprot/Q9MZU4 |
| Zinc finger protein 624 | **50** | 102.5 | 11 | **Q9P2J8** | http://www.uniprot.org/uniprot/Q9P2J8 |
| Neurofibromin | **56** | 320.5 | 16 | **P97526** | http://www.uniprot.org/uniprot/P97526 |
| Protein FAM184B | **54** | 121.9 | 13 | **Q9ULE4** | http://www.uniprot.org/uniprot/Q9ULE4 |
| Phosphomannomutase 2 | **58** | 28.4 | 6 | **Q3SZJ9** | http://www.uniprot.org/uniprot/Q3SZJ9 |
| Isocitrate dehydrogenase [NADP] cytoplasmic | **53** | 47 | 6 | **P41562** | http://www.uniprot.org/uniprot/P41562 |
| Beta-1,3-galactosyltransferase 4 | **44** | 42.9 | 7 | **Q5TJE8** | http://www.uniprot.org/uniprot/Q5TJE8 |
| Transmembrane protein 238 | **51** | 18.1 | 4 | **C9JI98** | http://www.uniprot.org/uniprot/C9JI98 |
| Protein FAM3C | **51** | 25.0 | 7 | **Q810F4** | http://www.uniprot.org/uniprot/Q810F4 |
| Coiled-coil domain-containing protein 136 | **51** | 133.7 | 9 | **Q3TVA9** | http://www.uniprot.org/uniprot/Q3TVA9 |
| Protein KHNYN | **50** | 75.1 | 9 | **Q80U38** | http://www.uniprot.org/uniprot/Q80U38 |
| Retinol-binding protein 2 | **50** | 15.8 | 4 | **Q08652** | http://www.uniprot.org/uniprot/Q08652 |
| Tetratricopeptide repeat protein 36 | **72** | 20.7 | 7 | **Q3SZV0** | http://www.uniprot.org/uniprot/Q3SZV0 |
| E3 ubiquitin-protein ligase RNF152 | **62** | 23.1 | 6 | **D2H6Z0** | http://www.uniprot.org/uniprot/D2H6Z0 |
| Protein RCC2 | **50** | 56.8 | 10 | **Q9P258** | http://www.uniprot.org/uniprot/Q9P258 |
| Signal peptidase complex subunit 2 | **54** | 25.3 | 6 | **Q5RAY6** | http://www.uniprot.org/uniprot/Q5RAY6 |
| Protein myomaker | **50** | 25.1 | 4 | **A6NI61** | http://www.uniprot.org/uniprot/A6NI61 |
| Apoptosis-enhancing nuclease | **57** | 37.6 | 9 | **Q9CZI9** | http://www.uniprot.org/uniprot/Q9CZI9 |
| Short-chain specific acyl-CoA dehydrogenase, mitochondrial | **53** | 44.6 | 9 | **P16219** | http://www.uniprot.org/uniprot/P16219 |
| Fanconi anemia group B protein | **61** | 99.4 | 12 | **Q8NB91** | http://www.uniprot.org/uniprot/Q8NB91 |
| Dual specificity phosphatase 28 | **70** | 18.7 | 6 | **Q4G0W2** | http://www.uniprot.org/uniprot/Q4G0W2 |
| Succinyl-CoA ligase [ADP-forming] subunit beta, mitochondrial | **65** | 50.3 | 12 | **Q4R517** | http://www.uniprot.org/uniprot/Q4R517 |
| Zinc finger and SCAN domain-containing protein 5A | **51** | 56.9 | 9 | **Q9BUG6** | http://www.uniprot.org/uniprot/Q9BUG6 |
| Eukaryotic translation initiation factor 4E-binding protein 1 | **50** | 12.7 | 5 | **Q0P5A7** | http://www.uniprot.org/uniprot/Q0P5A7 |
| 39S ribosomal protein L30, mitochondrial | **57** | 18.7 | 6 | **Q58DV5** | http://www.uniprot.org/uniprot/Q58DV5 |
| WAP four-disulfide core domain protein 12 | **57** | 12.7 | 4 | **A4K2P0** | http://www.uniprot.org/uniprot/A4K2P0 |
| UV-stimulated scaffold protein A | **55** | 82.6 | 12 | **Q9D479** | http://www.uniprot.org/uniprot/Q9D479 |
| NADH dehydrogenase [ubiquinone] 1 alpha subcomplex subunit 12 | **52** | 17.1 | 4 | **Q9UI09** | http://www.uniprot.org/uniprot/Q9UI09 |
| Tektin-4 | **59** | 51.3 | 8 | **Q8WW24** | http://www.uniprot.org/uniprot/Q8WW24 |
| Heterochromatin protein 1-binding protein 3 | **65** | 61.5 | 12 | **Q5SSJ5** | http://www.uniprot.org/uniprot/Q5SSJ5 |
| Natriuretic peptides B | **37** | 15.1 | 4 | **P16859** | http://www.uniprot.org/uniprot/P16859 |
| Zinc finger protein 624 | **59** | 102.5 | 12 | **Q9P2J8** | http://www.uniprot.org/uniprot/Q9P2J8 |
| G kinase-anchoring protein 1 | **55** | 42.2 | 5 | **Q5XIG5** | http://www.uniprot.org/uniprot/Q5XIG5 |
| OTU domain-containing protein 6B | **59** | 34 | 8 | **Q8N6M0** | http://www.uniprot.org/uniprot/Q8N6M0 |
| Tetratricopeptide repeat protein 36 | **64** | 20.7 | 9 | **Q3SZV0** | http://www.uniprot.org/uniprot/Q3SZV0 |
| Leucine-rich repeat and coiled-coil domain-containing protein 1 | **61** | 120.6 | 12 | **Q69ZB0** | http://www.uniprot.org/uniprot/Q69ZB0 |
| Poly [ADP-ribose] polymerase 12 | **61** | 80.5 | 12 | **Q9H0J9** | http://www.uniprot.org/uniprot/Q9H0J9 |
| Cortexin-2 | **53** | 9.1 | 6 | **P0C2S0** | http://www.uniprot.org/uniprot/P0C2S0 |
| Luc7-like protein 3 | **51** | 51.9 | 11 | **Q3SX41** | http://www.uniprot.org/uniprot/Q3SX41 |
| Putative uncharacterized protein encoded by CRHR1-IT1 | **52** | 17.2 | 9 | **Q96LR1** | http://www.uniprot.org/uniprot/Q96LR1 |
| DNA replication licensing factor MCM4 | **56** | 97.1 | 11 | **P33991** | http://www.uniprot.org/uniprot/P33991 |
| Acyl-CoA synthetase family member 2, mitochondrial | **63** | 69 | 9 | **Q17QJ1** | http://www.uniprot.org/uniprot/Q17QJ1 |
| Transthyretin | **45** | 16.4 | 3 | **P49143** | http://www.uniprot.org/uniprot/P49143] |
| Putative uncharacterized protein DKFZp434L187 | **62** | 15.1 | 8 | **Q9UFV3** | http://www.uniprot.org/uniprot/Q9UFV3 |
| Protein AAR2 homolog | **52** | 43.9 | 10 | **Q08DJ7** | http://www.uniprot.org/uniprot/Q08DJ7 |
| Annexin A5 | **55** | 36 | 5 | **P08758** | http://www.uniprot.org/uniprot/P08758 |
| Apolipoprotein A-II | **53** | 11.3 | 5 | **P0DN36** | http://www.uniprot.org/uniprot/P0DN36 |
| Retinol-binding protein 4 | **44** | 23.4 | 6 | **P27485** | http://www.uniprot.org/uniprot/P27485 |
| BTB/POZ domain-containing protein KCTD1 | **49** | 29.7 | 6 | **Q719H9** | http://www.uniprot.org/uniprot/Q719H9 |
| Collagen alpha-2(I) chain | **45** | 80.9 | 11 | **C0HJP6** | http://www.uniprot.org/uniprot/C0HJP6 |
| L-gulonolactone oxidase | **55** | 51 | 13 | **Q8HXW0** | http://www.uniprot.org/uniprot/Q8HXW0 |
| Zinc finger protein 624 | **58** | 102.5 | 12 | **Q9P2J8** | http://www.uniprot.org/uniprot/Q9P2J8 |
| Cilia- and flagella-associated protein 52 | **57** | 69.2 | 11 | **Q8N1V2** | http://www.uniprot.org/uniprot/Q8N1V2 |
| Autophagy-related protein 16-1 | **53** | 68.9 | 12 | **Q676U5** | http://www.uniprot.org/uniprot/Q676U5 |
| IQ domain-containing protein D | **55** | 51.8 | 13 | **Q17QH9** | http://www.uniprot.org/uniprot/Q17QH9 |
| Fibroblast growth factor 12 | **55** | 27.6 | 6 | **P61328** | http://www.uniprot.org/uniprot/P61328 |
| Interferon-induced protein with tetratricopeptide repeats 1 | **52** | 52.8 | 12 | **Q4R5F5** | http://www.uniprot.org/uniprot/Q4R5F5 |
| Fanconi anemia group B protein | **50** | 99.4 | 13 | **Q8NB91** | http://www.uniprot.org/uniprot/Q8NB91 |
| Protein TMEM155 | **55** | 14.4 | 5 | **Q5R4Y3** | http://www.uniprot.org/uniprot/Q5R4Y3 |
| Transmembrane protein 225 | **58** | 26.3 | 5 | **Q6GV28** | http://www.uniprot.org/uniprot/Q6GV28 |
| Telomerase reverse transcriptase | **65** | 128.6 | 10 | **O14746** | http://www.uniprot.org/uniprot/O14746 |
| Tetratricopeptide repeat protein 36 | **64** | 20.7 | 9 | **Q3SZV0** | http://www.uniprot.org/uniprot/Q3SZV0 |
| Arginine/serine-rich protein 1 | **52** | 33.7 | 8 | **Q9BUV0** | http://www.uniprot.org/uniprot/Q9BUV0 |
| V-set and transmembrane domain-containing  protein 2B | **61** | 30.4 | 6 | **A6NLU5** | http://www.uniprot.org/uniprot/A6NLU5 |
| Ninein | **52** | 245.2 | 20 | **Q8N4C6** | http://www.uniprot.org/uniprot/Q8N4C6 |
| Electron transfer flavoprotein subunit beta | **51** | 27.9 | 7 | **Q68FU3** | http://www.uniprot.org/uniprot/Q68FU3 |
| Coiled-coil domain-containing protein 25 | **53** | 24.6 | 6 | **Q86WR0** | http://www.uniprot.org/uniprot/Q86WR0 |
| Protein-arginine deiminase type-2 | **49** | 76 | 8 | **P20717** | http://www.uniprot.org/uniprot/P20717 |
| Radical S-adenosyl methionine domain-containing protein 2 | **55** | 42.4 | 8 | **Q9MZU4** | http://www.uniprot.org/uniprot/Q9MZU4 |
| Golgi SNAP receptor complex member 1 | **61** | 28.6 | 7 | **O88630** | http://www.uniprot.org/uniprot/O88630 |
| BTB/POZ domain-containing protein KCTD1 | **55** | 29.7 | 5 | **Q719H9** | http://www.uniprot.org/uniprot/Q719H9 |
| Zinc finger and SCAN domain-containing protein 5A | **57** | 56.9 | 9 | **Q9BUG6** | http://www.uniprot.org/uniprot/Q9BUG6 |
| Calcium-binding mitochondrial carrier protein SCaMC-3 | **51** | 52.7 | 9 | **Q6GQS1** | http://www.uniprot.org/uniprot/Q6GQS1 |
