# Supplementary material for: Urinary proteome of dogs with kidney injury during babesiosis
Source: BMC Vet Res. 2019 Dec 4;15:439. doi: 10.1186/s12917-019-2194-0 (PMC6894246; doi:10.1186/s12917-019-2194-0)
Supplement: Supplementary file 2 — Additional file 2 Table S2. Proteins identified in urine from dogs with babesiosis. [file 12917_2019_2194_MOESM2_ESM.docx]

| Table S2. Proteins identified in urine of dogs with babesiosis. | | | | | |
| --- | --- | --- | --- | --- | --- |
| Protein name | Score | Mass | Matches | Access no. | Hyperlink |
| Lipase member N | **54** | 45.7 | 8 | **Q5VXI9** | http://www.uniprot.org/uniprot/Q5VXI9 |
| Succinyl-CoA ligase [ADP-forming] subunit beta, mitochondrial | **57** | 50.3 | 9 | **Q4R517** | http://www.uniprot.org/uniprot/Q4R517 |
| TATA box-binding protein-like protein 2 | **51** | 39.3 | 5 | **Q6SJ95** | http://www.uniprot.org/uniprot/Q6SJ95 |
| Interferon gamma | **53** | 18.1 | 5 | **P01581** | http://www.uniprot.org/uniprot/P01581 |
| m7GpppX diphosphatase | **57** | 38.8 | 9 | **Q96C86** | http://www.uniprot.org/uniprot/Q96C86 |
| Peptidyl-prolyl cis-trans isomerase F, mitochondrial | **52** | 22. | 6 | **P30404** | http://www.uniprot.org/uniprot/P30404 |
| Mast cell carboxypeptidase A | **52** | 48.9 | 6 | **P15088** | http://www.uniprot.org/uniprot/P15088 |
| SprT-like domain-containing protein Spartan | **54** | 56.1 | 14 | **G3X912** | http://www.uniprot.org/uniprot/G3X912 |
| 39S ribosomal protein L50, mitochondrial | **54** | 18.3 | 9 | **Q8VDT9** | http://www.uniprot.org/uniprot/Q8VDT9 |
| Trafficking protein particle complex subunit 1 | **55** | 17 | 6 | **Q5NCF2** | http://www.uniprot.org/uniprot/Q5NCF2 |
| Desmin | **36** | 53.3 | 9 | **Q5XFN2** | http://www.uniprot.org/uniprot/Q5XFN2 |
| Dysferlin | **57** | 240 | 17 | **Q9ESD7** | http://www.uniprot.org/uniprot/Q9ESD7 |
| Leucine-rich repeat-containing protein 14 O | **54** | 55.7 | 6 | **A5PJJ5** | http://www.uniprot.org/uniprot/A5PJJ5 |
| Coenzyme Q-binding protein COQ10 homolog B, mitochondrial | **58** | 28 | 10 | **Q5I0I9** | http://www.uniprot.org/uniprot/Q5I0I9 |
| Bactericidal permeability-increasing protein (Fragment) | **54** | 49 | 6 | **Q28739** | http://www.uniprot.org/uniprot/Q28739 |
| G protein-activated inward rectifier potassium channel 4 | **51** | 48.3 | 6 | **P48548** | http://www.uniprot.org/uniprot/P48548 |
| Gamma-aminobutyric acid receptor subunit beta-2 | **56** | 59.3 | 11 | **P47870** | http://www.uniprot.org/uniprot/P47870 |
| Ankyrin repeat domain-containing protein 42 | **53** | 43.6 | 7 | **Q8N9B4** | http://www.uniprot.org/uniprot/Q8N9B4 |
| Protein FAM83B | **62** | 115.2 | 9 | **Q5T0W9** | http://www.uniprot.org/uniprot/Q5T0W9 |
| Gamma-aminobutyric acid receptor subunit alpha-4 | **62** | 61.3 | 8 | **Q9D6F4** | http://www.uniprot.org/uniprot/Q9D6F4 |
| Zinc finger protein 624 | **61** | 102.5 | 14 | **Q9P2J8** | http://www.uniprot.org/uniprot/Q9P2J8 |
| Hyaluronan and proteoglycan link protein 2 | **54** | 38.6 | 8 | **Q9ESM3** | http://www.uniprot.org/uniprot/Q9ESM3 |
| HAUS augmin-like complex subunit 2 | **55** | 23.4 | 4 | **Q5RE16** | http://www.uniprot.org/uniprot/Q5RE16 |
| Thromboxane-A synthase | **54** | 60.7 | 10 | **P49430** | http://www.uniprot.org/uniprot/P49430 |
| Vimentin | **54** | 53.7 | 12 | **P20152** | http://www.uniprot.org/uniprot/P20152 |
| TD and POZ domain-containing protein 2 | **51** | 42.2 | 10 | **Q717B2** | http://www.uniprot.org/uniprot/Q717B2 |
| Kinase suppressor of Ras 2 | **62** | 110 | 22 | Q3UVC0 | http://www.uniprot.org/uniprot/Q3UVC0 |
| Cortexin-2 | **55** | 9.2 | 8 | **Q3URE8** | http://www.uniprot.org/uniprot/Q3URE8 |
| Transmembrane protein 240 | **53** | 20.3 | 5 | **Q5SV17** | http://www.uniprot.org/uniprot/Q5SV17 |
| Protein FAM71C | **50** | 27.9 | 11 | **Q8NEG0** | http://www.uniprot.org/uniprot/Q8NEG0 |
| Carboxylesterase 1E | **58** | 61.8 | 13 | **Q64176** | http://www.uniprot.org/uniprot/Q64176 |
| Cytochrome P450 3A31 | **67** | 58 | 13 | **O70537** | http://www.uniprot.org/uniprot/O70537 |
| Leucine-rich repeat-containing protein 14 | **61** | 55.3 | 6 | Q15048 | http://www.uniprot.org/uniprot/Q15048 |
| Protein phosphatase Slingshot homolog 1 | **66** | 116.5 | 15 | Q8WYL5 | http://www.uniprot.org/uniprot/Q8WYL5 |
| Twinkle protein, mitochondrial | **53** | 77.6 | 8 | **Q96RR1** | http://www.uniprot.org/uniprot/Q96RR1 |
| Ubiquitin-conjugating enzyme E2 N | **53** | 17.2 | 4 | **Q0P5K3** | http://www.uniprot.org/uniprot/Q0P5K3 |
| Delta-1-pyrroline-5-carboxylate synthase | **55** | 87.8 | 7 | **Q9Z110** | http://www.uniprot.org/uniprot/Q9Z110 |
| 40S ribosomal protein S11 | **35** | 18.6 | 3 | Q9XSU4 | http://www.uniprot.org/uniprot/Q9XSU4 |
| Synaptic vesicle membrane protein VAT-1 homolog-like | **61** | 46.2 | 8 | Q9HCJ6 | http://www.uniprot.org/uniprot/Q9HCJ6 |
| Glutamyl-tRNA(Gln) amidotransferase subunit C, mitochondrial | **40** | 18 | 6 | E2RK33 | http://www.uniprot.org/uniprot/E2RK33 |
| Trafficking protein particle complex subunit 1 | **63** | 17 | 7 | Q5NCF2 | http://www.uniprot.org/uniprot/Q5NCF2 |
| Rab GDP dissociation inhibitor beta | **44** | 50.8 | 8 | O97556 | http://www.uniprot.org/uniprot/O97556 |
| Parvalbumin alpha | **54** | 12.1 | 10 | **P20472** | http://www.uniprot.org/uniprot/P20472 |
| B-cell lymphoma 6 protein homolog | **52** | 58.8 | 11 | **P41183** | http://www.uniprot.org/uniprot/P41183 |
| Probable tubulin polyglutamylase TTLL1 | **53** | 49.5 | 9 | **Q5PPI9** | http://www.uniprot.org/uniprot/Q5PPI9 |
| Neurofilament medium polypeptide | **50** | 95.8 | 7 | **P12839** | http://www.uniprot.org/uniprot/P12839 |
| Suppressor of tumorigenicity 7 protein | **55** | 67.7 | 9 | **Q07E08** | http://www.uniprot.org/uniprot/Q07E08 |
| Vesicle transport protein USE1 | **67** | 30.8 | 7 | **Q9CQ56** | http://www.uniprot.org/uniprot/Q9CQ56 |
| Protein C12orf4 homolog | **67** | 54.3 | 8 | **D4A770** | http://www.uniprot.org/uniprot/D4A770 |
| Cell death activator CIDE-A | **67** | 24.8 | 6 | **O70302** | http://www.uniprot.org/uniprot/O70302 |
| Tryptophan 5-hydroxylase 2 | **58** | 56.8 | 9 | **Q2HZ26** | http://www.uniprot.org/uniprot/Q2HZ26 |
| Kelch-like protein | **57** | 70.2 | 8 | **Q2T9Z7** | http://www.uniprot.org/uniprot/Q2T9Z7 |
| Ubiquitin carboxyl-terminal hydrolase 37 | **63** | 111 | 12 | **Q86T82** | http://www.uniprot.org/uniprot/Q86T82 |
| Coatomer subunit beta' | **60** | 103.2 | 6 | **P35605** | http://www.uniprot.org/uniprot/P35605 |
| Centrosomal protein of 170 kDa protein B | **61** | 171.2 | 11 | **Q80U49** | http://www.uniprot.org/uniprot/Q80U49 |
| Eukaryotic translation initiation factor 4 gamma 2 | **68** | 102.6 | 15 | **Q62448** | http://www.uniprot.org/uniprot/Q62448 |
| Trifunctional enzyme subunit alpha, mitochondrial | **51** | 83.3 | 6 | **Q64428** | http://www.uniprot.org/uniprot/Q64428 |
| Isovaleryl-CoA dehydrogenase, mitochondrial | **55** | 46.9 | 6 | **P12007** | http://www.uniprot.org/uniprot/P12007 |
| Pericentrin | **60** | 380.6 | 32 | **O95613** | http://www.uniprot.org/uniprot/O95613 |
| Hydroxysteroid dehydrogenase-like protein 2 | **54** | 45.5 | 5 | **A4FUZ6** | http://www.uniprot.org/uniprot/A4FUZ6 |
| Ras-related protein Rab-34, isoform NARR | **53** | 21.1 | 4 | **P0DI83** | http://www.uniprot.org/uniprot/P0DI83 |
| Interferon-induced protein with tetratricopeptide repeats 1 | **70** | 55.8 | 11 | **Q4R5F5** | http://www.uniprot.org/uniprot/Q4R5F5 |
| Cysteine--tRNA ligase, mitochondrial | **62** | 62 | 12 | **Q2KIF8** | http://www.uniprot.org/uniprot/Q2KIF8 |
| Ubiquitin carboxyl-terminal hydrolase 37 | **63** | 111.2 | 13 | **F1N5V1** | http://www.uniprot.org/uniprot/F1N5V1 |
| Interferon regulatory factor 2-binding protein 1 | **67** | 62.6 | 8 | **Q8IU81** | http://www.uniprot.org/uniprot/Q8IU81 |
| Ubiquitin carboxyl-terminal hydrolase 37 | **84** | 111.2 | 14 | **F1N5V1** | http://www.uniprot.org/uniprot/F1N5V1 |
| Centrosomal protein of 152 kDa | **55** | 197.9 | 12 | **O94986** | http://www.uniprot.org/uniprot/O94986 |
| E3 SUMO-protein ligase PIAS2 | **57** | 64.3 | 9 | **Q6AZ28** | http://www.uniprot.org/uniprot/Q6AZ28 |
| ATR-interacting protein | **61** | 72.4 | 11 | **Q9N077** | http://www.uniprot.org/uniprot/Q9N077 |
| T-cell surface glycoprotein CD3 epsilon chain | **54** | 23 | 4 | **P27597** | http://www.uniprot.org/uniprot/P27597 |
| Rab GDP dissociation inhibitor beta | **55** | 50.8 | 10 | **O97556** | http://www.uniprot.org/uniprot/O97556 |
| Elongation factor Tu GTP-binding domain-containing protein 1 | **64** | 127.1 | 16 | **Q8C0D5** | http://www.uniprot.org/uniprot/Q8C0D5 |
| HIV Tat-specific factor 1 homolog | **57** | 86.6 | 12 | **Q8BGC0** | http://www.uniprot.org/uniprot/Q8BGC0 |
| Peptidyl-prolyl cis-trans isomerase A | **58** | 18.1 | 5 | **Q9TTC6** | http://www.uniprot.org/uniprot/Q9TTC6 |
| Tektin-4 | **58** | 51.3 | 7 | **Q8WW24** | http://www.uniprot.org/uniprot/Q8WW24 |
| Sp110 nuclear body protein | **58** | 79.6 | 9 | **Q9HB58** | http://www.uniprot.org/uniprot/Q9HB58 |
| Rab11 family-interacting protein 5 | **57** | 69.9 | 10 | **Q8R361** | http://www.uniprot.org/uniprot/Q8R361 |
| Alkyldihydroxyacetonephosphate synthase, peroxisomal | **53** | 73.7 | 8 | **O00116** | http://www.uniprot.org/uniprot/O00116 |
| Fibroblast growth factor 9 | **57** | 23.5 | 4 | **P31371** | http://www.uniprot.org/uniprot/P31371 |
| Lysozyme C | **52** | 16.9 | 5 | **Q659U0** | http://www.uniprot.org/uniprot/Q659U0 |
| Phosphoribosyl pyrophosphate synthase-associated protein 2 | **55** | 41.2 | 7 | **O08618** | http://www.uniprot.org/uniprot/O08618 |
| Zona pellucida sperm-binding protein 3 | **60** | 47.1 | 6 | **P42098** | http://www.uniprot.org/uniprot/P42098 |
| Galactoside 2-alpha-L-fucosyltransferase 2 | **62** | 39.1 | 8 | **O77485** | http://www.uniprot.org/uniprot/O77485 |
| 39S ribosomal protein L30, mitochondrial | **56** | 18.7 | 7 | **Q58DV5** | http://www.uniprot.org/uniprot/Q58DV5 |
| Desmin | **47** | 53.3 | 6 | **Q5XFN2** | http://www.uniprot.org/uniprot/Q5XFN2 |
| Dipeptidyl peptidase 1 (Fragment) | **43** | 50.1 | 4 | **O97578** | http://www.uniprot.org/uniprot/O97578 |
| Vimentin | **50** | 53.7 | 8 | **P20152** | http://www.uniprot.org/uniprot/P20152 |
| Serine/threonine-protein kinase 3 | **53** | 57.1 | 9 | **Q9JI10** | http://www.uniprot.org/uniprot/Q9JI10 |
| Neurofilament medium polypeptide | **62** | 95.8 | 12 | **P12839** | http://www.uniprot.org/uniprot/P12839 |
| Rab GDP dissociation inhibitor beta | **62** | 50.8 | 8 | **O97556** | http://www.uniprot.org/uniprot/O97556 |
| Zinc finger protein 624 | **65** | 102.5 | 10 | **Q9P2J8** | http://www.uniprot.org/uniprot/Q9P2J8 |
| Uncharacterized protein KIAA1683 homolog | **64** | 87 | 6 | **Q8WNU4** | http://www.uniprot.org/uniprot/Q8WNU4 |
| Phosphatidylserine decarboxylase proenzyme | **56** | 47.7 | 5 | **Q58DH2** | http://www.uniprot.org/uniprot/Q58DH2 |
| Probable tubulin polyglutamylase TTLL1 | **66** | 49.4 | 9 | **Q0VC71** | http://www.uniprot.org/uniprot/Q0VC71 |
| ATP synthase subunit d, mitochondrial | **63** | 18.7 | 5 | **P13620** | http://www.uniprot.org/uniprot/P13620 |
| Breast cancer anti-estrogen resistance protein 3 | **61** | 93.5 | 8 | **Q58DL5** | http://www.uniprot.org/uniprot/Q58DL5 |
| Sperm surface protein Sp17 | **61** | 17.4 | 4 | **Q15506** | http://www.uniprot.org/uniprot/Q15506 |
| Actin-related protein T1 | **55** | 42.1 | 6 | **Q4R821** | http://www.uniprot.org/uniprot/Q4R821 |
| Inhibitor of nuclear factor kappa-B kinase subunit beta | **54** | 87.8 | 7 | **O88351** | http://www.uniprot.org/uniprot/O88351 |
| Glucosamine-6-phosphate isomerase 2 | **53** | 31.3 | 5 | **Q9CRC9** | http://www.uniprot.org/uniprot/Q9CRC9 |
| E3 ubiquitin-protein ligase TRIM32 | **55** | 73.5 | 9 | **Q13049** | http://www.uniprot.org/uniprot/Q13049 |
| Protein FAM98B | **56** | 45.9 | 6 | **Q80VD1** | http://www.uniprot.org/uniprot/Q80VD1 |
| DNA topoisomerase 2-alpha | **56** | 173.5 | 11 | **Q01320** | http://www.uniprot.org/uniprot/Q01320 |
| Interferon alpha-1/13 | **50** | 22.1 | 4 | **P01562** | http://www.uniprot.org/uniprot/P01562 |
| Bone morphogenetic protein 7 | **56** | 21.5 | 6 | **P34819** | http://www.uniprot.org/uniprot/P34819 |
| Protein phosphatase Slingshot homolog 1 | **60** | 116.5 | 16 | **Q8WYL5** | http://www.uniprot.org/uniprot/Q8WYL5 |
| Serum albumin | **42** | 70.6 | 14 | **P49822** | http://www.uniprot.org/uniprot/P49822 |
| Peroxiredoxin-1 | **61** | 22.3 | 9 | **Q6B4U9** | http://www.uniprot.org/uniprot/Q6B4U9 |
| Zinc finger BED domain-containing protein 5 | **48** | 80.2 | 11 | **A4Z944** | http://www.uniprot.org/uniprot/A4Z944 |
| Zinc finger and SCAN domain-containing protein 9 | **57** | 47 | 9 | **O15535** | http://www.uniprot.org/uniprot/O15535 |
| NADH dehydrogenase [ubiquinone] 1 alpha subcomplex subunit 2 | **69** | 11.1 | 7 | **Q4R5E2** | http://www.uniprot.org/uniprot/Q4R5E2 |
| Tektin-4 | **67** | 51.3 | 11 | **Q8WW24** | http://www.uniprot.org/uniprot/Q8WW24 |
| Elongation factor Tu GTP-binding domain-containing protein 1 | **70** | 127.1 | 16 | **Q8C0D5** | http://www.uniprot.org/uniprot/Q8C0D5 |
| DCC-interacting protein 13-beta | **68** | 75 | 7 | **Q8NEU8** | http://www.uniprot.org/uniprot/Q8NEU8 |
| Ras-related protein Rab-28 | **55** | 25 | 5 | **Q3SWY9** | http://www.uniprot.org/uniprot/Q3SWY9 |
| Translation initiation factor eIF-2B subunit delta | **55** | 58.4 | 8 | **Q63186** | http://www.uniprot.org/uniprot/Q63186 |
| D-beta-hydroxybutyrate dehydrogenase, mitochondrial | **62** | 15.2 | 7 | **P86198** | http://www.uniprot.org/uniprot/P86198 |
| Gap junction alpha-8 protein | **69** | 49.9 | 12 | **Q8K4Q9** | http://www.uniprot.org/uniprot/Q8K4Q9 |
| Dual specificity phosphatase 28 | **54** | 18.7 | 7 | **Q4G0W2** | http://www.uniprot.org/uniprot/Q4G0W2 |
| 39S ribosomal protein L30, mitochondrial | **53** | 18.7 | 7 | **Q58DV5** | http://www.uniprot.org/uniprot/Q58DV5 |
| Ras-related protein Rab-36 | **61** | 36.8 | 12 | **O95755** | http://www.uniprot.org/uniprot/O95755 |
| tRNA-dihydrouridine(20) synthase [NAD(P)+]-like | **50** | 55.8 | 10 | **Q9NX74** | http://www.uniprot.org/uniprot/Q9NX74 |
| AP-3 complex subunit mu-2 | **55** | 47.2 | 5 | **P53677** | http://www.uniprot.org/uniprot/P53677 |
| OTU domain-containing protein 6B | **52** | 34 | 7 | **Q8N6M0** | http://www.uniprot.org/uniprot/Q8N6M0 |
| A-kinase anchor protein 10, mitochondrial | **57** | 74.1 | 10 | **O88845** | http://www.uniprot.org/uniprot/O88845 |
| Hemoglobin subunit beta | **56** | 16.3 | 6 | **P02073** | http://www.uniprot.org/uniprot/P02073 |
| Single-pass membrane and coiled-coil domain-containing protein 2 | **55** | 34.1 | 8 | **Q95JR4** | http://www.uniprot.org/uniprot/Q95JR4 |
| Myotrophin | **56** | 13.1 | 5 | **Q3T0F7** | http://www.uniprot.org/uniprot/Q3T0F7 |
| Zinc finger protein 622 | **77** | 54.8 | 17 | **Q969S3** | http://www.uniprot.org/uniprot/Q969S3 |
| Protein POF1B | **63** | 68.9 | 10 | **Q8WVV4** | http://www.uniprot.org/uniprot/Q8WVV4 |
| HORMA domain-containing protein 1 | **57** | 45.4 | 8 | **D3ZWE7** | http://www.uniprot.org/uniprot/D3ZWE7 |
| Sperm surface protein Sp17 | **58** | 17.4 | 7 | **Q15506** | http://www.uniprot.org/uniprot/Q15506 |
| Tyrosine-protein kinase BAZ1B | **56** | 172.2 | 14 | **Q9Z277** | http://www.uniprot.org/uniprot/Q9Z277 |
| Nicolin-1 | **43** | 24.6 | 8 | **Q861Y6** | http://www.uniprot.org/uniprot/Q861Y6 |
| Parvalbumin alpha | **63** | 12.1 | 11 | **P20472** | http://www.uniprot.org/uniprot/P20472 |
| Vimentin (Fragment) | **58** | 51.9 | 12 | **P48670** | http://www.uniprot.org/uniprot/P48670 |
| Protein FAM3C | **52** | 24.9 | 7 | **Q92520** | http://www.uniprot.org/uniprot/Q92520 |
| Uncharacterized aarF domain-containing protein kinase 5 | **53** | 66.3 | 8 | **Q3MIX3** | http://www.uniprot.org/uniprot/Q3MIX3 |
| Interferon-induced protein with tetratricopeptide repeats 1 | **51** | 55.8 | 9 | **P09914** | http://www.uniprot.org/uniprot/P09914 |
| Oxidoreductase HTATIP2 | **50** | 27.4 | 10 | **A2T7G9** | http://www.uniprot.org/uniprot/A2T7G9 |
| Glutathione S-transferase alpha M14 | **52** | 25.4 | 10 | **GSTA1_PIG** | http://www.uniprot.org/uniprot/P51781 |
| Sperm acrosome membrane-associated protein 3 | **50** | 18.8 | 8 | **B6VH75** | http://www.uniprot.org/uniprot/B6VH75 |
| Protein FAM204A | **53** | 27.1 | 6 | **Q8C6C7** | http://www.uniprot.org/uniprot/Q8C6C7 |
| Ras-related protein Rab-36 | **55** | 36.8 | 8 | **O95755** | http://www.uniprot.org/uniprot/O95755 |
| Neurofibromin | **50** | 323.1 | 24 | **Q04690** | http://www.uniprot.org/uniprot/Q04690 |
| Oxidoreductase HTATIP2 | **52** | 27.4 | 6 | **A2T7G9** | http://www.uniprot.org/uniprot/A2T7G9 |
| Transmembrane emp24 domain-containing protein 9 | **61** | 27.5 | 6 | **Q3T133** | http://www.uniprot.org/uniprot/Q3T133 |
| Centromere protein H | **61** | 28 | 8 | **Q3T0L1** | http://www.uniprot.org/uniprot/Q3T0L1 |
| Centrosomal protein of 104 kDa | **72** | 105 | 14 | **Q80V31** | http://www.uniprot.org/uniprot/Q80V31 |
| Nicolin-1 | **55** | 24.5 | 6 | **Q9BSH3** | http://www.uniprot.org/uniprot/Q9BSH3 |
| Acylphosphatase-2 | **57** | 10.9 | 5 | **P35745** | http://www.uniprot.org/uniprot/P35745 |
| Mitofusin-1 | **58** | 84.5 | 9 | **Q811U4** | http://www.uniprot.org/uniprot/Q811U4 |
| Ras-related protein Rab-36 | **54** | 36.8 | 9 | **O95755** | http://www.uniprot.org/uniprot/O95755 |
| Hippocalcin-like protein 1 | **51** | 22.4 | 7 | **P62748** | http://www.uniprot.org/uniprot/P62748 |
| Stanniocalcin-2 | **47** | 34.1 | 9 | **Q5RAT2** | http://www.uniprot.org/uniprot/Q5RAT2 |
| Major vault protein | **51** | 96.2 | 14 | **Q9EQK5** | http://www.uniprot.org/uniprot/Q9EQK5 |
| Peptidyl-prolyl cis-trans isomerase FKBP1A | **59** | 12 | 4 | **Q62658** | http://www.uniprot.org/uniprot/Q62658 |
| Tyrosine-protein phosphatase non-receptor type 12 | **54** | 87.2 | 17 | **P35831** | http://www.uniprot.org/uniprot/P35831 |
| Kinase suppressor of Ras 2 | **52** | 108.9 | 16 | **Q6VAB6** | http://www.uniprot.org/uniprot/Q6VAB6 |
| Putative ATP-dependent RNA helicase DHX30 | **54** | 136.9 | 10 | **Q2NKY8** | http://www.uniprot.org/uniprot/Q2NKY8 |
| U3 small nucleolar RNA-associated protein 14 homolog A | **53** | 88.2 | 10 | **Q3T0Q8** | http://www.uniprot.org/uniprot/Q3T0Q8 |
| 39S ribosomal protein L30, mitochondrial | **64** | 18.7 | 7 | **Q58DV5** | http://www.uniprot.org/uniprot/Q58DV5 |
| Growth arrest and DNA damage-inducible proteins-interacting protein 1 | **63** | 25.9 | 8 | **Q9CR59** | http://www.uniprot.org/uniprot/Q9CR59 |
| Protein FAM57B | **66** | 31.2 | 7 | **Q71RH2** | http://www.uniprot.org/uniprot/Q71RH2 |
| Ras-related protein Rab-9A | **35** | 23.1 | 4 | **P24408** | http://www.uniprot.org/uniprot/P24408 |
| Probable tubulin polyglutamylase TTLL1 | **53** | 49.5 | 6 | **Q5PPI9** | http://www.uniprot.org/uniprot/Q5PPI9 |
| Adenylyl cyclase-associated protein 2 | **51** | 53.1 | 8 | **Q9CYT6** | http://www.uniprot.org/uniprot/Q9CYT6 |
| Collagen alpha-1(I) chain (Fragments) | **55** | 75.2 | 12 | **C0HJP1** | http://www.uniprot.org/uniprot/C0HJP1 |
| NADH dehydrogenase [ubiquinone] flavoprotein 1, mitochondrial | **54** | 51.4 | 8 | **Q0MQI4** | http://www.uniprot.org/uniprot/Q0MQI4 |
| Rho GTPase-activating protein 18 | **54** | 75.2 | 9 | **Q8N392** | http://www.uniprot.org/uniprot/Q8N392 |
| Rho GTPase-activating protein 39 | **65** | 122.2 | 11 | **Q9C0H5** | http://www.uniprot.org/uniprot/Q9C0H5 |
| Unconventional myosin-Ie | **71** | 127.4 | 13 | **Q63356** | http://www.uniprot.org/uniprot/Q63356 |
| Zinc finger and SCAN domain-containing protein 25 | **55** | 62.6 | 8 | **Q6NSZ9** | http://www.uniprot.org/uniprot/Q6NSZ9 |
| Four and a half LIM domains protein 2 | **55** | 34.1 | 5 | **O35115** | http://www.uniprot.org/uniprot/O35115 |
| Putative uncharacterized protein MYH16 | **59** | 128,4 | 16 | **Q9H6N6** | http://www.uniprot.org/uniprot/Q9H6N6 |
| EGF domain-specific O-linked N-acetylglucosamine transferase | **61** | 62.6 | 12 | **A0JND3** | http://www.uniprot.org/uniprot/A0JND3 |
| Annexin A10 | **55** | 37.8 | 6 | **Q9UJ72** | http://www.uniprot.org/uniprot/Q9UJ72 |
| Beta-crystallin B2 | **51** | 37.8 | 4 | **P02522** | http://www.uniprot.org/uniprot/P02522 |
| Tripartite motif-containing protein 42 | **51** | 85.7 | 8 | **Q8IWZ5** | http://www.uniprot.org/uniprot/Q8IWZ5 |
| Syndecan-4 | **53** | 21.5 | 4 | **O35988** | http://www.uniprot.org/uniprot/O35988 |
| OTU domain-containing protein 6B | **53** | 34 | 7 | **Q8N6M0** | http://www.uniprot.org/uniprot/Q8N6M0 |
| Desmin | **65** | 53.6 | 7 | **O62654** | http://www.uniprot.org/uniprot/O62654 |
| Cytospin-A | **50** | 125 | 10 | **Q2KNA0** | http://www.uniprot.org/uniprot/Q2KNA0 |
| OTU domain-containing protein 6B | **53** | 34 | 7 | **Q8N6M0** | http://www.uniprot.org/uniprot/Q8N6M0 |
| Glutathione S-transferase alpha M14 | **66** | 25.4 | 6 | **P51781** | http://www.uniprot.org/uniprot/P51781 |
| Ribosome-binding protein 1 | **53** | 164.8 | 13 | **Q28298** | http://www.uniprot.org/uniprot/Q28298 |
| Zinc finger protein castor homolog 1 | **56** | 193.3 | 18 | **Q86V15** | http://www.uniprot.org/uniprot/Q86V15 |
| Sorting nexin-3 | **53** | 18.8 | 6 | **Q1RMH8** | http://www.uniprot.org/uniprot/Q1RMH8 |
| Succinate dehydrogenase [ubiquinone] iron-sulfur subunit, mitochondrial | **61** | 32.6 | 6 | **Q9CQA3** | http://www.uniprot.org/uniprot/Q9CQA3 |
| Coiled-coil domain-containing protein 122 | **60** | 32.4 | 7 | **Q5T0U0** | http://www.uniprot.org/uniprot/Q5T0U0 |
| Zinc finger C2HC domain-containing protein 1C | **58** | 61.1 | 15 | **Q9BGW4** | http://www.uniprot.org/uniprot/Q9BGW4 |
| Ceramide synthase 3 | **58** | 61.1 | 8 | **Q8IU89** | http://www.uniprot.org/uniprot/Q8IU89 |
| Tetratricopeptide repeat protein 36 | **55** | 20.7 | 8 | **Q3SZV0** | http://www.uniprot.org/uniprot/Q3SZV0 |
| Ras-related protein Rab-17 | **51** | 23.7 | 9 | **Q9H0T7** | http://www.uniprot.org/uniprot/Q9H0T7 |
| Transmembrane and coiled-coil domain-containing protein 5A | **52** | 34.5 | 5 | **Q8N6Q1** | http://www.uniprot.org/uniprot/Q8N6Q1 |
| Inosine triphosphate pyrophosphatase | **56** | 21.8 | 6 | **Q9BY32** | http://www.uniprot.org/uniprot/Q9BY32 |
| Vacuolar protein sorting-associated protein 29 | **51** | 20.7 | 6 | **Q9UBQ0** | http://www.uniprot.org/uniprot/Q9UBQ0 |
| AP-3 complex subunit mu-2 | **66** | 47.2 | 8 | **P53677** | http://www.uniprot.org/uniprot/P53677 |
| Ras and EF-hand domain-containing protein homolog | **60** | 71.3 | 10 | **Q5RI75** | http://www.uniprot.org/uniprot/Q5RI75 |
| Protein C19orf12 homolog | **65** | 15.1 | 6 | **Q8WUR0** | http://www.uniprot.org/uniprot/Q8WUR0 |
| Serine palmitoyltransferase 2 | **54** | 63.6 | 9 | **O15270** | http://www.uniprot.org/uniprot/O15270 |
| Thioredoxin, mitochondrial | **53** | 18.4 | 4 | **P97493** | http://www.uniprot.org/uniprot/P97493 |
| Vesicle transport protein USE1 | **57** | 30.8 | 9 | **Q9CQ56** | http://www.uniprot.org/uniprot/Q9CQ56 |
| Far upstream element-binding protein 3 O | **55** | 61.9 | 7 | **Q96I24** | http://www.uniprot.org/uniprot/Q96I24 |
| Nesprin-3 | **51** | 112.3 | 20 | **Q4FZC9** | http://www.uniprot.org/uniprot/Q4FZC9 |
| Zinc finger protein 532 | **50** | 112.2 | 15 | **Q6NXK2** | http://www.uniprot.org/uniprot/Q6NXK2 |
| Hemoglobin subunit epsilon (Fragment) | **50** | 15.6 | 6 | **O13071** | http://www.uniprot.org/uniprot/O13071 |
| Vimentin | **60** | 53.7 | 15 | **P20152** | http://www.uniprot.org/uniprot/P20152 |
| Centromere protein H | **61** | 28 | 7 | **Q3T0L1** | http://www.uniprot.org/uniprot/Q3T0L1 |
| Potassium/sodium hyperpolarization-activated cyclic nucleotide-gated channel 1 | **60** | 93.4 | 11 | **Q9MZS1** | http://www.uniprot.org/uniprot/Q9MZS1 |
| Pleckstrin homology-like domain family A member 3 | **59** | 13.9 | 5 | **Q9WV95** | http://www.uniprot.org/uniprot/Q9WV95 |
| Zinc finger and SCAN domain-containing protein 5A | **71** | 56.9 | 11 | **Q9BUG6** | http://www.uniprot.org/uniprot/Q9BUG6 |
| Signal recognition particle receptor subunit beta | **55** | 29.7 | 7 | **Q4FZX7** | http://www.uniprot.org/uniprot/Q4FZX7 |
| Tetratricopeptide repeat protein 6 | **53** | 60 | 8 | **Q86TZ1** | http://www.uniprot.org/uniprot/Q86TZ1 |
| Protein phosphatase 1 regulatory subunit 36 | **53** | 47.7 | 8 | **D3Z0R2** | http://www.uniprot.org/uniprot/D3Z0R2 |
| Oculomedin | **53** | 5.3 | 4 | **Q9Y5M6** | http://www.uniprot.org/uniprot/Q9Y5M6 |
| Interleukin-2 | **57** | 17.8 | 6 | **Q95KP3** | http://www.uniprot.org/uniprot/Q95KP3 |
| NADH dehydrogenase [ubiquinone] 1 alpha subcomplex subunit 2 | **54** | 11 | 5 | **O43678** | http://www.uniprot.org/uniprot/O43678 |
| Ras-related protein Rab-36 | **57** | 36.8 | 7 | **O95755** | http://www.uniprot.org/uniprot/O95755 |
| ATP-dependent 6-phosphofructokinase, liver type | **51** | 58.8 | 10 | **P17858** | http://www.uniprot.org/uniprot/P17858 |
| AarF domain-containing protein kinase 4 | **52** | 59.6 | 6 | **Q6AY19** | http://www.uniprot.org/uniprot/Q6AY19 |
| Fidgetin-like protein 1 | **61** | 74.8 | 12 | **Q6PIW4** | http://www.uniprot.org/uniprot/Q6PIW4 |
| Neutrophil cytosol factor 4 | **61** | 39.1 | 8 | **Q15080** | http://www.uniprot.org/uniprot/Q15080 |
| Glycine amidinotransferase, mitochondrial | **61** | 48.8 | 4 | **Q9D964** | http://www.uniprot.org/uniprot/Q9D964 |
| Uncharacterized protein C1orf168 homolog | **62** | 82.8 | 9 | **A2A995** | http://www.uniprot.org/uniprot/A2A995 |
| Haptoglobin | **51** | 36.9 | 5 | **P19006** | http://www.uniprot.org/uniprot/P19006 |
| F-box/LRR-repeat protein 8 | **53** | 41.4 | 5 | **Q96CD0** | http://www.uniprot.org/uniprot/Q96CD0 |
| Desmin | **45** | 53.3 | 9 | **Q5XFN2** | http://www.uniprot.org/uniprot/Q5XFN2 |
| Torsin-4A | **52** | 47.3 | 6 | **Q9NXH8** | http://www.uniprot.org/uniprot/Q9NXH8 |
| OTU domain-containing protein 6B | **61** | 34 | 6 | **Q8N6M0** | http://www.uniprot.org/uniprot/Q8N6M0 |
| 39S ribosomal protein L2, mitochondrial | **63** | 33.5 | 9 | **Q2TA12** | http://www.uniprot.org/uniprot/Q2TA12 |
| Telomerase reverse transcriptase | **61** | 128.6 | 8 | **O14746** | http://www.uniprot.org/uniprot/O14746 |
| Solute carrier family 15 member 1 | **48** | 79.3 | 5 | **Q8WMX5** | http://www.uniprot.org/uniprot/Q8WMX5 |
| Tripartite motif-containing protein 75 | **59** | 54.4 | 7 | **Q3UWZ0** | http://www.uniprot.org/uniprot/Q3UWZ0 |
| Probable ATP-dependent RNA helicase DDX28 | **59** | 59.8 | 6 | **Q9NUL7** | http://www.uniprot.org/uniprot/Q9NUL7 |
| Glutamyl-tRNA(Gln) amidotransferase subunit C, mitochondrial | **57** | 17.6 | 4 | **E2RK33** | http://www.uniprot.org/uniprot/E2RK33 |
| Cap-specific mRNA (nucleoside-2'-O-)-methyltransferase 1 | **59** | 96.6 | 12 | **Q9DBC3** | http://www.uniprot.org/uniprot/Q9DBC3 |
| Protein MB21D2 | **65** | 49 | 8 | **Q8C525** | http://www.uniprot.org/uniprot/Q8C525 |
| Desmin | **29** | 53.3 | 3 | **Q5XFN2** | http://www.uniprot.org/uniprot/Q5XFN2 |
| NADH dehydrogenase [ubiquinone] 1 alpha subcomplex subunit 2 | **60** | 11 | 5 | **O43678** | http://www.uniprot.org/uniprot/O43678 |
| Oculomedin | **46** | 5.3 | 4 | **Q9Y5M6** | http://www.uniprot.org/uniprot/Q9Y5M6 |
| Glycogen phosphorylase, brain form | **52** | 96.9 | 12 | **Q5MIB6** | http://www.uniprot.org/uniprot/Q5MIB6 |
| V-set and transmembrane domain-containing protein 2B | **62** | 30.3 | 5 | **Q9JME9** | http://www.uniprot.org/uniprot/Q9JME9 |
| Microsomal triglyceride transfer protein large subunit | **65** | 99.6 | 11 | **P55156** | http://www.uniprot.org/uniprot/P55156 |
| E3 ubiquitin-protein ligase ARIH2 | **56** | 59.3 | 8 | **Q9Z1K6** | http://www.uniprot.org/uniprot/Q9Z1K6 |
| Ubiquitin carboxyl-terminal hydrolase 37 | **58** | 111 | 6 | **Q86T82** | http://www.uniprot.org/uniprot/Q86T82 |
| Putative homeodomain transcription factor 1 | **53** | 88 | 9 | **Q9UMS5** | http://www.uniprot.org/uniprot/Q9UMS5 |
| Junction plakoglobin | **53** | 82.4 | 6 | **P14923** | http://www.uniprot.org/uniprot/P14923 |
| Pericentriolar material 1 protein | **52** | 230 | 10 | **Q15154** | http://www.uniprot.org/uniprot/Q15154 |
| 43 kDa receptor-associated protein of the synapse | **60** | 47.6 | 13 | **P12672** | http://www.uniprot.org/uniprot/P12672 |
| Desmin | **45** | 53.3 | 6 | **Q5XFN2** | http://www.uniprot.org/uniprot/Q5XFN2 |
| Probable tubulin polyglutamylase TTLL1 | **63** | 49.4 | 10 | **O95922** | http://www.uniprot.org/uniprot/O95922 |
| Ubiquitin carboxyl-terminal hydrolase 48 | **61** | 121.1 | 8 | **Q86UV5** | http://www.uniprot.org/uniprot/Q86UV5 |
| Parvalbumin alpha | **62** | 12.1 | 7 | **P20472** | http://www.uniprot.org/uniprot/P20472 |
| Putative fatty acid-binding protein 5-like protein 3 | **51** | 11.5 | 6 | **A8MUU1** | http://www.uniprot.org/uniprot/A8MUU1 |
| Tyrosine-protein phosphatase non-receptor type 6 | **49** | 70 | 11 | **P81718** | http://www.uniprot.org/uniprot/P81718 |
| Zinc finger protein 624 | **65** | 102.5 | 13 | **Q9P2J8** | http://www.uniprot.org/uniprot/Q9P2J8 |
| Zinc finger and SCAN domain-containing protein 5A | **65** | 56.9 | 11 | **Q9BUG6** | http://www.uniprot.org/uniprot/Q9BUG6 |
| Actin-related protein 2/3 complex subunit 3 | **51** | 20.8 | 7 | **Q3T035** | http://www.uniprot.org/uniprot/Q3T035 |
| FSD1-like protein | **56** | 60.2 | 9 | **Q9BXM9** | http://www.uniprot.org/uniprot/Q9BXM9 |
| UPF0585 protein C16orf13 | **54** | 22.8 | 7 | **Q96S19** | http://www.uniprot.org/uniprot/Q96S19 |
| HAUS augmin-like complex subunit 6 | **60** | 109.6 | 12 | **Q7Z4H7** | http://www.uniprot.org/uniprot/Q7Z4H7 |
| FSD1-like protein | **62** | 60.2 | 9 | **Q9BXM9** | http://www.uniprot.org/uniprot/Q9BXM9 |
| Protocadherin alpha-C2 | **62** | 110 | 8 | **Q9Y5I4** | http://www.uniprot.org/uniprot/Q9Y5I4 |
| Ral guanine nucleotide dissociation stimulator-like 2 | **63** | 84.5 | 7 | **Q5TJE5** | http://www.uniprot.org/uniprot/Q5TJE5 |
| Cilia- and flagella-associated protein 99 | **57** | 52.5 | 7 | **D6REC4** | http://www.uniprot.org/uniprot/D6REC4 |
| Ras-related protein Rab-39A | **51** | 25.4 | 5 | **Q14964** | http://www.uniprot.org/uniprot/Q14964 |
| Protein FAM229B | **63** | 8.9 | 5 | **Q4G0N7** | http://www.uniprot.org/uniprot/Q4G0N7 |
| Zinc finger protein ZFP69B | **55** | 62.8 | 6 | **Q9UJL9** | http://www.uniprot.org/uniprot/Q9UJL9 |
| NAD-dependent protein deacetylase sirtuin-7 | **53** | 45.7 | 7 | **B2RZ55** | http://www.uniprot.org/uniprot/B2RZ55 |
| Zona pellucida sperm-binding protein 2 | **44** | 81.1 | 8 | **P47983** | http://www.uniprot.org/uniprot/P47983 |
| Peroxiredoxin-1 | **61** | 22.3 | 7 | **Q06830** | http://www.uniprot.org/uniprot/Q06830 |
| Transcriptional repressor NF-X1 | **54** | 130 | 10 | **Q12986** | http://www.uniprot.org/uniprot/Q12986 |
| Phosphatidylinositol 4-phosphate 5-kinase type-1 alpha | **62** | 60.8 | 12 | **P70182** | http://www.uniprot.org/uniprot/P70182 |
| Transcriptional repressor NF-X1 | **53** | 130 | 10 | **P70182** | http://www.uniprot.org/uniprot/P70182 |
| Tyrosine-protein kinase receptor TYRO3 | **52** | 97.2 | 8 | **P55144** | http://www.uniprot.org/uniprot/P55144 |
| Ras-related protein Rab-17 | **53** | 23.7 | 5 | **Q9H0T7** | http://www.uniprot.org/uniprot/Q9H0T7 |
| Thiamine-triphosphatase | **54** | 25.7 | 9 | **Q9BU02** | http://www.uniprot.org/uniprot/Q9BU02 |
| Dual specificity phosphatase 28 | **52** | 18.7 | 7 | **Q4G0W2** | http://www.uniprot.org/uniprot/Q4G0W2 |
| Peptidyl-prolyl cis-trans isomerase D | **57** | 41.1 | 8 | **Q9CR16** | http://www.uniprot.org/uniprot/Q9CR16 |
| Artemin | **51** | 24.2 | 6 | **Q6AYE8** | http://www.uniprot.org/uniprot/Q6AYE8 |
| Zinc finger and SCAN domain-containing protein 5A | **77** | 56.9 | 7 | **Q9BUG6** | http://www.uniprot.org/uniprot/Q9BUG6 |
| SLAIN motif-containing protein 2 | **50** | 62.6 | 7 | **Q8CI08** | http://www.uniprot.org/uniprot/Q8CI08 |
| Adenylyl cyclase-associated protein 2 | **52** | 53.2 | 7 | **P52481** | http://www.uniprot.org/uniprot/P52481 |
| Acylphosphatase-2 | **51** | 11.2 | 6 | **P00818** | http://www.uniprot.org/uniprot/P00818 |
| Centriolar coiled-coil protein of 110 kDa | **51** | 11.9 | 14 | **Q7TSH4** | http://www.uniprot.org/uniprot/Q7TSH4 |
| Zinc finger and SCAN domain-containing protein 5A | **51** | 56.9 | 8 | **Q9BUG6** | http://www.uniprot.org/uniprot/Q9BUG6 |
| Fibronectin type 3 and ankyrin repeat domains protein 1 | **54** | 38.6 | 6 | **Q6B858** | http://www.uniprot.org/uniprot/Q6B858 |
| Hemoglobin subunit beta | **60** | 16.3 | 8 | **Q6B858** | http://www.uniprot.org/uniprot/Q6B858 |
| Ankyrin repeat and death domain-containing protein 1A | **51** | 57.9 | 10 | **Q495B1** | http://www.uniprot.org/uniprot/Q495B1 |
| Protein-arginine deiminase type-4 | **63** | 75.1 | 11 | **Q9Z183** | http://www.uniprot.org/uniprot/Q9Z183 |
| Actin-binding Rho-activating protein | **60** | 43 | 10 | **Q8BUZ1** | http://www.uniprot.org/uniprot/Q8BUZ1 |
| Myotrophin | **51** | 13.1 | 6 | **Q3T0F7** | http://www.uniprot.org/uniprot/Q3T0F7 |
| Ras GTPase-activating protein 2 | **52** | 97.8 | 9 | **P58069** | http://www.uniprot.org/uniprot/P58069 |
| Cleavage and polyadenylation specificity factor subunit 1 | **54** | 162.4 | 9 | **Q10569** | http://www.uniprot.org/uniprot/Q10569 |
| Cholecystokinin | **55** | 12.9 | 5 | **P41520** | http://www.uniprot.org/uniprot/P41520 |
| Cortexin-2 | **55** | 9.2 | 4 | **Q3URE8** | http://www.uniprot.org/uniprot/Q3URE8 |
| Uncharacterized protein C12orf60 | **53** | 27.7 | 7 | **Q5U649** | http://www.uniprot.org/uniprot/Q5U649 |
| Tetratricopeptide repeat protein 36 | **66** | 20.7 | 9 | **Q3SZV0** | http://www.uniprot.org/uniprot/Q3SZV0 |
| Coiled-coil domain-containing protein 81 | **64** | 76.8 | 18 | **Q6ZN84** | http://www.uniprot.org/uniprot/Q6ZN84 |
| Tyrosine-protein kinase Fer | **63** | 95.1 | 23 | **Q9TTY2** | http://www.uniprot.org/uniprot/Q9TTY2 |
| Tetratricopeptide repeat protein 36 | **59** | 20.7 | 8 | **Q3SZV0** | http://www.uniprot.org/uniprot/Q3SZV0 |
| Ras-related protein Rab-36 | **64** | 36.8 | 10 | **O95755** | http://www.uniprot.org/uniprot/O95755 |
| Rab GDP dissociation inhibitor beta | **66** | 50.8 | 8 | **O97556** | http://www.uniprot.org/uniprot/O97556 |
| Centromere/kinetochore protein zw10 homolog | **63** | 89.6 | 15 | **O43264** | http://www.uniprot.org/uniprot/O43264 |
| Parvalbumin alpha | **62** | 12.1 | 10 | **P20472** | http://www.uniprot.org/uniprot/P20472 |
| Complement C1q subcomponent subunit B | **62** | 26.6 | 8 | **Q2KIV9** | http://www.uniprot.org/uniprot/Q2KIV9 |
| Double-stranded RNA-binding protein Staufen homolog 2 | **68** | 62.8 | 10 | **Q9NUL3** | http://www.uniprot.org/uniprot/Q9NUL3 |
| CD209 antigen-like protein B | **66** | 37.7 | 7 | **Q8CJ91** | http://www.uniprot.org/uniprot/Q8CJ91 |
| SRC kinase signaling inhibitor 1 | **66** | 112.7 | 11 | **Q9C0H9** | http://www.uniprot.org/uniprot/Q9C0H9 |
| Oxidoreductase HTATIP2 | **72** | 27.4 | 7 | **A2T7G9** | http://www.uniprot.org/uniprot/A2T7G9 |
| Tyrosine--tRNA ligase, cytoplasmic | **51** | 59.4 | 12 | **Q4KM49** | http://www.uniprot.org/uniprot/Q4KM49 |
| Zinc finger protein 101 | **50** | 51.9 | 10 | **Q8IZC7** | http://www.uniprot.org/uniprot/Q8IZC7 |
| 60S ribosomal protein L37 | **53** | 11.3 | 7 | **P79244** | http://www.uniprot.org/uniprot/P79244 |
| Zinc finger protein 621 | **52** | 50.2 | 6 | **Q6ZSS3** | http://www.uniprot.org/uniprot/Q6ZSS3 |
| E3 ubiquitin-protein ligase RNF152 | **50** | 23.1 | 5 | **D2H6Z0** | http://www.uniprot.org/uniprot/D2H6Z0 |
| Ras-related protein Rab-36 | **68** | 36.8 | 9 | **O95755** | http://www.uniprot.org/uniprot/O95755 |
| Prolactin | **64** | 26.1 | 7 | **Q9QZL1** | http://www.uniprot.org/uniprot/Q9QZL1 |
| Vacuolar ATPase assembly integral membrane protein Vma21 | **50** | 44.4 | 4 | **Q78T54** | http://www.uniprot.org/uniprot/Q78T54 |
| Hemoglobin subunit beta | **69** | 16.3 | 10 | **P02073** | http://www.uniprot.org/uniprot/P02073 |
| Sterile alpha and TIR motif-containing protein 1 | **60** | 80.8 | 12 | **I3L5V6** | http://www.uniprot.org/uniprot/I3L5V6 |
| 39S ribosomal protein L50, mitochondrial | **62** | 18.3 | 6 | **Q8VDT9** | http://www.uniprot.org/uniprot/Q8VDT9 |
| Interferon regulatory factor 2-binding protein 1 | **66** | 62.6 | 14 | **Q8IU81** | http://www.uniprot.org/uniprot/Q8IU81 |
| FUN14 domain-containing protein 2 | **50** | 16.6 | 5 | **Q9D6K8** | http://www.uniprot.org/uniprot/Q9D6K8 |
| Potassium voltage-gated channel subfamily B member 1 | **62** | 96.7 | 13 | **Q14721** | http://www.uniprot.org/uniprot/Q14721 |
| Collagen alpha-2(I) chain | **64** | 129.8 | 9 | **O46392** | http://www.uniprot.org/uniprot/O46392 |
| Protein AAR2 homolog | **61** | 43.9 | 9 | **Q08DJ7** | http://www.uniprot.org/uniprot/Q08DJ7 |
| Aurora kinase C | **65** | 35.9 | 13 | **Q9UQB9** | http://www.uniprot.org/uniprot/Q9UQB9 |
| NAD-dependent protein deacetylase sirtuin-7 | **58** | 45.7 | 14 | **Q8BKJ9** | http://www.uniprot.org/uniprot/Q8BKJ9 |
| Peroxiredoxin-1 | **56** | 22.3 | 9 | **Q06830** | http://www.uniprot.org/uniprot/Q06830 |
| Perilipin-3 | **52** | 47.1 | 7 | **Q5RAV8** | http://www.uniprot.org/uniprot/Q5RAV8 |
| Protein kish-A | **52** | 8.3 | 4 | **Q148I3** | http://www.uniprot.org/uniprot/Q148I3 |
| Ras-related protein Rab-36 | **50** | 36.8 | 6 | **O95755** | http://www.uniprot.org/uniprot/O95755 |
| Succinyl-CoA ligase [ADP-forming] subunit beta, mitochondrial | **62** | 50.3 | 12 | **Q4R517** | http://www.uniprot.org/uniprot/Q4R517 |
| Glycogen phosphorylase, liver form | **51** | 97.9 | 12 | **P09811** | http://www.uniprot.org/uniprot/P09811 |
| Integrin alpha-11 | **50** | 134.1 | 13 | **P61622** | http://www.uniprot.org/uniprot/P61622 |
| Neuropathy target esterase | **63** | 150.9 | 17 | **Q3TRM4** | http://www.uniprot.org/uniprot/Q3TRM4 |
| Oncostatin-M | **61** | 28.8 | 9 | **P13725** | http://www.uniprot.org/uniprot/P13725 |
| Vesicle transport protein USE1 | **63** | 30.8 | 9 | **Q9CQ56** | http://www.uniprot.org/uniprot/Q9CQ56 |
| Testicular haploid expressed gene protein | **50** | 43.8 | 8 | **Q5XHX8** | http://www.uniprot.org/uniprot/Q5XHX8 |
| Serine/threonine-protein phosphatase 6 catalytic subunit | **55** | 35.8 | 7 | **O00743** | http://www.uniprot.org/uniprot/O00743 |
| E3 ubiquitin-protein ligase MARCH8 | **53** | 33.7 | 5 | **Q0VD59** | http://www.uniprot.org/uniprot/Q0VD59 |
| Phospholipase A2 | **25** | 17.0 | 3 | **P06596** | http://www.uniprot.org/uniprot/P06596 |
| Protein polyglycylase TTLL10 | **55** | 80.2 | 13 | **A4Q9F3** | http://www.uniprot.org/uniprot/A4Q9F3 |
| T-complex protein 1 subunit gamma | **58** | 61.1 | 13 | **Q3T0K2** | http://www.uniprot.org/uniprot/Q3T0K2 |
| Vimentin (Fragment) | **57** | 51.9 | 14 | **P48670** | http://www.uniprot.org/uniprot/P48670 |
| Ras-related protein Rab-36 | **61** | 36.8 | 9 | **O95755** | http://www.uniprot.org/uniprot/O95755 |
| Interferon-induced protein with tetratricopeptide repeats 1 | **54** | 55.8 | 10 | **Q4R5F5** | http://www.uniprot.org/uniprot/Q4R5F5 |
| Uncharacterized protein C1orf186 | **52** | 19.6 | 4 | **Q6ZWK4** | http://www.uniprot.org/uniprot/Q6ZWK4 |
| Isocitrate dehydrogenase [NADP] cytoplasmic | **51** | 47.1 | 7 | **Q9XSG3** | http://www.uniprot.org/uniprot/Q9XSG3 |
| Dual specificity phosphatase | **51** | 18.7 | 5 | **Q4G0W2** | http://www.uniprot.org/uniprot/Q4G0W2 |
| Methylmalonic aciduria type A protein, mitochondrial | **50** | 46.9 | 6 | **Q8IVH4** | http://www.uniprot.org/uniprot/Q8IVH4 |
| Rab GDP dissociation inhibitor beta | **47** | 50.8 | 8 | **O97556** | http://www.uniprot.org/uniprot/O97556 |
| Vacuolar protein sorting-associated protein 4B | **62** | 49.5 | 14 | **Q5R658** | http://www.uniprot.org/uniprot/Q5R658 |
| C-type natriuretic peptide | **66** | 13.5 | 9 | **P56283** | http://www.uniprot.org/uniprot/P56283 |
| T-complex protein 1 subunit alpha | **64** | 60.8 | 14 | **P18279** | http://www.uniprot.org/uniprot/P18279 |
| Carboxylesterase 1E | **67** | 61.8 | 11 | **Q64176** | http://www.uniprot.org/uniprot/Q64176 |
| Stefin-2 | **52** | 11.9 | 6 | **P35174** | http://www.uniprot.org/uniprot/P35174 |
| Ras-related protein Rab-2B | **55** | 24.5 | 6 | **P59279** | http://www.uniprot.org/uniprot/P59279 |
| Hemoglobin subunit epsilon (Fragment) | **53** | 15.6 | 5 | **O13071** | http://www.uniprot.org/uniprot/O13071 |
| Adenylosuccinate synthetase lisozyme 1 | **53** | 50.5 | 8 | **Q8N142** | http://www.uniprot.org/uniprot/Q8N142 |
| Fragile X mental retardation protein 1 homolog | **52** | 67.3 | 7 | **Q5R9B4** | http://www.uniprot.org/uniprot/Q5R9B4 |
| Calponin-1 | **50** | 33.4 | 8 | **Q2HJ38** | http://www.uniprot.org/uniprot/Q2HJ38 |
| Calponin-1 | **84** | 33.4 | 10 | **Q9GK38** | http://www.uniprot.org/uniprot/Q9GK38 |
| Tyrosine-tRNA ligase, cytoplasmic | **58** | 59.5 | 9 | **Q5R8T5** | http://www.uniprot.org/uniprot/Q5R8T5 |
| 14 kDa phosphohistidine phosphatase | **50** | 14 | 4 | **Q9NRX4** | http://www.uniprot.org/uniprot/Q9NRX4 |
| N-acetylgalactosamine-6-sulfatase | **45** | 58.4 | 4 | **Q32KH5** | http://www.uniprot.org/uniprot/Q32KH5 |
| Norrin | **58** | 15.6 | 6 | **Q2KI78** | http://www.uniprot.org/uniprot/Q2KI78 |
| Ribosome biogenesis protein BOP1 | **50** | 83.2 | 8 | **P97452** | http://www.uniprot.org/uniprot/P97452 |
| Sp110 nuclear body protein | **51** | 79.6 | 7 | **Q9HB58** | http://www.uniprot.org/uniprot/Q9HB58 |
| Endophilin-A2 | **52** | 41.7 | 7 | **Q62419** | http://www.uniprot.org/uniprot/Q62419 |
| Ras-related protein Rab-36 | **54** | 36.8 | 5 | **O95755** | http://www.uniprot.org/uniprot/O95755 |
| Putative uncharacterized protein C6orf50 | **53** | 12.6 | 5 | **Q9HD87** | http://www.uniprot.org/uniprot/Q9HD87 |
| Cytochrome P450 2C23 | **52** | 57 | 8 | **P24470** | http://www.uniprot.org/uniprot/P24470 |
| Protein MB21D2 | **52** | 49 | 9 | **Q8C525** | http://www.uniprot.org/uniprot/Q8C525 |
| Heat shock 70 kDa protein 4L (Fragments) | **52** | 23.8 | 9 | **P86265** | http://www.uniprot.org/uniprot/P86265 |
| Oxidoreductase HTATIP2 | **53** | 27.4 | 6 | **A2T7G9** | http://www.uniprot.org/uniprot/A2T7G9 |
| Ras-related protein Rab-36 | **56** | 36.8 | 6 | **O95755** | http://www.uniprot.org/uniprot/O95755 |
| OTU domain-containing protein 6B | **54** | 34 | 7 | **Q8N6M0** | http://www.uniprot.org/uniprot/Q8N6M0 |
| Suppressor of IKBKE 1 | **53** | 23.7 | 8 | **Q9CPR7** | http://www.uniprot.org/uniprot/Q9CPR7 |
| Myb/SANT-like DNA-binding domain-containing protein 3 | **64** | 32.7 | 8 | **Q0III0** | http://www.uniprot.org/uniprot/Q0III0 |
| Ras-related protein Rab-36 | **52** | 36.8 | 8 | **O95755** | http://www.uniprot.org/uniprot/O95755 |
| Calpastatin | **50** | 77.6 | 11 | **P27321** | http://www.uniprot.org/uniprot/P27321 |
| NACHT, LRR and PYD domains-containing protein 5 | **62** | 123.4 | 21 | **Q647I9** | http://www.uniprot.org/uniprot/Q647I9 |
| Mitochondrial uncoupling protein 3 | **38** | 34.6 | 5 | **Q9N2I9** | http://www.uniprot.org/uniprot/Q9N2I9 |
| Hemoglobin subunit beta | **62** | 16.3 | 8 | **P02073** | http://www.uniprot.org/uniprot/P02073 |
| Diacylglycerol kinase theta | **50** | 104 | 11 | **Q6P5E8** | http://www.uniprot.org/uniprot/Q6P5E8 |
| C-type natriuretic peptide | **65** | 13.5 | 8 | **P56283** | http://www.uniprot.org/uniprot/P56283 |
| Interleukin-12 receptor subunit beta-2 | **55** | 98.5 | 17 | **Q99665** | http://www.uniprot.org/uniprot/Q99665 |
| Tumor necrosis factor receptor superfamily member 5 | **58** | 33.4 | 7 | **P27512** | http://www.uniprot.org/uniprot/P27512 |
| Tetratricopeptide repeat protein 34 | **53** | 61.8 | 10 | **A8MYJ7** | http://www.uniprot.org/uniprot/A8MYJ7 |
| TATA box-binding protein-associated factor RNA polymerase I subunit D | **52** | 33 | 4 | **Q5M948** | http://www.uniprot.org/uniprot/Q5M948 |
| Tetratricopeptide repeat protein 36 | **65** | 20.7 | 9 | **Q3SZV0** | http://www.uniprot.org/uniprot/Q3SZV0 |
| Nicolin-1 | **62** | 24.5 | 9 | **Q9BSH3** | http://www.uniprot.org/uniprot/Q9BSH3 |
| Gamma-tubulin complex component 2 | **51** | 103.8 | 22 | **Q921G8** | http://www.uniprot.org/uniprot/Q921G8 |
| Calcium/calmodulin-dependent protein kinase II inhibitor 1 | **52** | 8.6 | 6 | **A7MBG3** | http://www.uniprot.org/uniprot/A7MBG3 |
| Tropomodulin-4 | **53** | 39.5 | 7 | **Q0VC48** | http://www.uniprot.org/uniprot/Q0VC48 |
| Protein THEM6 | **51** | 24 | 6 | **Q5XIE1** | http://www.uniprot.org/uniprot/Q5XIE1 |
| F-box/SPRY domain-containing protein 1 | **55** | 31.1 | 7 | **Q8K3B1** | http://www.uniprot.org/uniprot/Q8K3B1 |
| Actin-like protein 7B | **52** | 45.9 | 9 | **Q9Y614** | http://www.uniprot.org/uniprot/Q9Y614 |
| Kinase suppressor of Ras 2 | **56** | 110 | 20 | **Q3UVC0** | http://www.uniprot.org/uniprot/Q3UVC0 |
| Cytospin-A | **50** | 125 | 17 | **Q2KNA0** | http://www.uniprot.org/uniprot/Q2KNA0 |
| Uridine 5'-monophosphate synthase | **51** | 52.6 | 8 | **P11172** | http://www.uniprot.org/uniprot/P11172 |
| Coiled-coil domain-containing protein 25 | **50** | 24.6 | 8 | **Q78PG9** | http://www.uniprot.org/uniprot/Q78PG9 |
| NADH dehydrogenase [ubiquinone] 1 alpha subcomplex subunit 12 | **64** | 17.1 | 6 | **Q9UI09** | http://www.uniprot.org/uniprot/Q9UI09 |
| Ras-related protein Rab-36 | **62** | 36.8 | 9 | **O95755** | http://www.uniprot.org/uniprot/O95755 |
| Epididymal-specific lipocalin-5 | **65** | 20.8 | 8 | **P06911** | http://www.uniprot.org/uniprot/P06911 |
| NADH dehydrogenase [ubiquinone] 1 alpha subcomplex subunit 2 | **57** | 11.1 | 7 | **Q4R5E2** | http://www.uniprot.org/uniprot/Q4R5E2 |
| Zinc finger protein castor homolog 1 | **72** | 193.3 | 15 | **Q86V15** | http://www.uniprot.org/uniprot/Q86V15 |
| Hippocalcin-like protein 1 | **62** | 22.4 | 6 | **P62748** | http://www.uniprot.org/uniprot/P62748 |
| Uncharacterized protein C12orf60 | **56** | 27.7 | 10 | **Q5U649** | http://www.uniprot.org/uniprot/Q5U649 |
| Mini-chromosome maintenance complex-binding protein | **56** | 73.6 | 10 | **Q8R3C0** | http://www.uniprot.org/uniprot/Q8R3C0 |
| Transmembrane protein 132C | **59** | 122.6 | 8 | **Q8N3T6** | http://www.uniprot.org/uniprot/Q8N3T6 |
| Ubiquitin carboxyl-terminal hydrolase isozyme L1 | **56** | 25.2 | 5 | **P50103** | http://www.uniprot.org/uniprot/P50103 |
| Apolipoprotein A-II | **54** | 11.2 | 4 | **P0DM93** | http://www.uniprot.org/uniprot/P0DM93 |
| Kinesin light chain 2 | **52** | 69.3 | 10 | **Q9H0B6** | http://www.uniprot.org/uniprot/Q9H0B6 |
| Glutathione peroxidase 3 | **50** | 25.6 | 6 | **P23764** | http://www.uniprot.org/uniprot/P23764 |
| Prolactin | **55** | 26.6 | 5 | **P12420** | http://www.uniprot.org/uniprot/P12420 |
| Arginine/serine-rich protein 1 | **51** | 33.7 | 5 | **Q9BUV0** | http://www.uniprot.org/uniprot/Q9BUV0 |
| Amyloid beta A4 protein (Fragment) | **58** | 6.2 | 4 | **Q29149** | http://www.uniprot.org/uniprot/Q29149 |
| Leucine-rich repeat-containing protein 49 | **61** | 79.4 | 7 | **Q91YK0** | http://www.uniprot.org/uniprot/Q91YK0 |
| PH and SEC7 domain-containing protein 3 | **50** | 115.3 | 11 | **Q2PFD7** | http://www.uniprot.org/uniprot/Q2PFD7 |
| Insulin | **54** | 12.5 | 5 | **P01321** | http://www.uniprot.org/uniprot/P01321 |
| Dual specificity phosphatase 28 | **54** | 18.7 | 6 | **Q4G0W2** | http://www.uniprot.org/uniprot/Q4G0W2 |
| Calpain-2 catalytic subunit | **50** | 80.8 | 8 | **P17655** | http://www.uniprot.org/uniprot/P17655 |
| Peroxiredoxin-1 | **52** | 22.3 | 6 | **Q06830** | http://www.uniprot.org/uniprot/Q06830 |
| Ras-related protein Rab-25 | **53** | 23.5 | 6 | **P46629** | http://www.uniprot.org/uniprot/P46629 |
| tRNA-dihydrouridine(47) synthase [NAD(P)(+)]-like | **53** | 72.4 | 9 | **Q91XI1** | http://www.uniprot.org/uniprot/Q91XI1 |
| AarF domain-containing protein kinase 4 | **56** | 59.6 | 8 | **Q6AY19** | http://www.uniprot.org/uniprot/Q6AY19 |
| RING finger protein 10 | **52** | 90.7 | 7 | **Q08E13** | http://www.uniprot.org/uniprot/Q08E13 |
| E3 ubiquitin-protein ligase RNF152 | **60** | 23.1 | 5 | **D2H6Z0** | http://www.uniprot.org/uniprot/D2H6Z0 |
| Protein-arginine deiminase type-3 | **53** | 76.3 | 7 | **Q9Z184** | http://www.uniprot.org/uniprot/Q9Z184 |
| Sentan | **65** | 16.6 | 5 | **A6NMZ2** | http://www.uniprot.org/uniprot/A6NMZ2 |
| Plakophilin-4 | **53** | 132.3 | 8 | **Q68FH0** | http://www.uniprot.org/uniprot/Q68FH0 |
| Urotensin-2B | **65** | 13.0 | 8 | **Q765I1** | http://www.uniprot.org/uniprot/Q765I1 |
| Signal peptidase complex subunit 2 | **53** | 25.3 | 8 | **Q15005** | http://www.uniprot.org/uniprot/Q15005 |
| Tektin-4 | **54** | 51.3 | 12 | **Q8WW24** | http://www.uniprot.org/uniprot/Q8WW24 |
| Cytochrome P450 2J3 | **53** | 58.4 | 10 | **P51590** | http://www.uniprot.org/uniprot/P51590 |
| Ras-related protein Rab-36 | **64** | 36.8 | 9 | **O95755** | http://www.uniprot.org/uniprot/O95755 |
| Isocitrate dehydrogenase [NADP] cytoplasmic | **50** | 47.2 | 6 | **Q6XUZ5** | http://www.uniprot.org/uniprot/Q6XUZ5 |
| E3 ubiquitin-protein ligase RNF169 | **55** | 77.1 | 8 | **E9Q7F2** | http://www.uniprot.org/uniprot/E9Q7F2 |
| Class E basic helix-loop-helix protein 40 | **51** | 45.9 | 6 | **O14503** | http://www.uniprot.org/uniprot/O14503 |
| Interleukin-13 | **55** | 15.5 | 6 | **Q9N0W9** | http://www.uniprot.org/uniprot/Q9N0W9 |
| Rab GDP dissociation inhibitor beta | **67** | 50.8 | 7 | **O97556** | http://www.uniprot.org/uniprot/O97556 |
| Isocitrate dehydrogenase [NADP], mitochondrial | **60** | 51.3 | 8 | **P54071** | http://www.uniprot.org/uniprot/P54071 |
| Stanniocalcin-2 | **63** | 34.1 | 8 | **O97561** | http://www.uniprot.org/uniprot/O97561 |
| Ephrin type-B receptor 4 | **50** | 110.3 | 11 | **P54761** | http://www.uniprot.org/uniprot/P54761 |
| NADH dehydrogenase [ubiquinone] 1 alpha subcomplex subunit 12 | **60** | 17.1 | 7 | **Q9UI09** | http://www.uniprot.org/uniprot/Q9UI09 |
| Dual specificity phosphatase 28 | **54** | 18.7 | 7 | **Q4G0W2** | http://www.uniprot.org/uniprot/Q4G0W2 |
| Parafibromin | **54** | 60.7 | 13 | **Q6P1J9** | http://www.uniprot.org/uniprot/Q6P1J9 |
| Bactericidal permeability-increasing protein | **59** | 54 | 10 | **Q6AXU0** | http://www.uniprot.org/uniprot/Q6AXU0 |
| Zinc finger protein castor homolog 1 | **61** | 193.3 | 17 | **Q86V15** | http://www.uniprot.org/uniprot/Q86V15 |
| Calcitonin receptor-stimulating peptide 2 | **44** | 14.3 | 7 | **Q75V93** | http://www.uniprot.org/uniprot/Q75V93 |
| Corticoliberin | **58** | 20.8 | 9 | **Q95MI6** | http://www.uniprot.org/uniprot/Q95MI6 |
| Urotensin-2B | **50** | 13 | 6 | **Q765I1** | http://www.uniprot.org/uniprot/Q765I1 |
| Pumilio homolog 3 | **53** | 73.9 | 16 | **Q15397** | http://www.uniprot.org/uniprot/Q15397 |
| Protein FAM162B | **55** | 18.1 | 9 | **A6QPI4** | http://www.uniprot.org/uniprot/A6QPI4 |
| Cytochrome c oxidase subunit 6C | **55** | 8.6 | 7 | **Q7YRK2** | http://www.uniprot.org/uniprot/Q7YRK2 |
| Aromatase | **56** | 58.6 | 13 | **P46194** | http://www.uniprot.org/uniprot/P46194 |
| Zinc finger protein 101 | **50** | 51.9 | 9 | **Q8IZC7** | http://www.uniprot.org/uniprot/Q8IZC7 |
| Phosphatidylethanolamine-binding protein 1 | **52** | 21.2 | 5 | **Q8MK67** | http://www.uniprot.org/uniprot/Q8MK67 |
| Leucine-tRNA ligase, cytoplasmic | **57** | 135.7 | 11 | **Q5R614** | http://www.uniprot.org/uniprot/Q5R614 |
| NACHT, LRR and PYD domains-containing protein 10 | **76** | 77.3 | 14 | **Q8CCN1** | http://www.uniprot.org/uniprot/Q8CCN1 |
| Urotensin-2B | **70** | 13 | 7 | **Q765I1** | http://www.uniprot.org/uniprot/Q765I1 |
| Mortality factor 4-like protein 2 | **82** | 32.2 | 8 | **Q9R0Q4** | http://www.uniprot.org/uniprot/Q9R0Q4 |
| Nucleolar pre-ribosomal-associated protein 1 | **59** | 256.5 | 15 | **O60287** | http://www.uniprot.org/uniprot/O60287 |
| Serine/threonine-protein phosphatase 6 catalytic subunit | **74** | 35.8 | 10 | **O00743** | http://www.uniprot.org/uniprot/O00743 |
| Hemopexin | **63** | 52 | 7 | **Q91X72** | http://www.uniprot.org/uniprot/Q91X72 |
| Rab GDP dissociation inhibitor beta | **52** | 51 | 8 | **P50397** | http://www.uniprot.org/uniprot/P50397 |
| Sperm surface protein Sp17 | **55** | 17.3 | 5 | **Q62252** | http://www.uniprot.org/uniprot/Q62252 |
| Natriuretic peptides B | **46** | 15.1 | 4 | **P16859** | http://www.uniprot.org/uniprot/P16859 |
| Iron-responsive element-binding protein 2 | **55** | 106.1 | 8 | **B3VKQ2** | http://www.uniprot.org/uniprot/B3VKQ2 |
| STE20-related kinase adapter protein alpha | **53** | 41.9 | 5 | **Q5E9J9** | http://www.uniprot.org/uniprot/Q5E9J9 |
| E3 ubiquitin-protein ligase RNF169 | **50** | 77.1 | 9 | **E9Q7F2** | http://www.uniprot.org/uniprot/E9Q7F2 |
| Acyl-coenzyme A synthetase ACSM2B, mitochondrial | **57** | 64.8 | 8 | **Q68CK6** | http://www.uniprot.org/uniprot/Q68CK6 |
| Survival motor neuron protein | **63** | 32.2 | 9 | **Q4R4F8** | http://www.uniprot.org/uniprot/Q4R4F8 |
| Tektin-4 | **54** | 51.3 | 8 | **Q8WW24** | http://www.uniprot.org/uniprot/Q8WW24 |
| Anaphase-promoting complex subunit CDC26 | **61** | 9.8 | 4 | **Q3SZT7** | http://www.uniprot.org/uniprot/Q3SZT7 |
| 39S ribosomal protein L30, mitochondrial | **71** | 18.7 | 5 | **Q58DV5** | http://www.uniprot.org/uniprot/Q58DV5 |
| Interleukin-4 | **64** | 15.5 | 6 | **P55030** | http://www.uniprot.org/uniprot/P55030 |
| 39S ribosomal protein L10, mitochondrial | **51** | 29.6 | 8 | **Q3TBW2** | http://www.uniprot.org/uniprot/Q3TBW2 |
| 39S ribosomal protein L30, mitochondrial | **50** | 18.7 | 5 | **Q58DV5** | http://www.uniprot.org/uniprot/Q58DV5 |
| Acylphosphatase-2 | **61** | 11.1 | 7 | **P35744** | http://www.uniprot.org/uniprot/P35744 |
| Tektin-4 | **51** | 51.3 | 8 | **Q8WW24** | http://www.uniprot.org/uniprot/Q8WW24 |
| Potassium/sodium hyperpolarization-activated cyclic nucleotide-gated channel 2 | **68** | 95.7 | 17 | **Q9JKA9** | http://www.uniprot.org/uniprot/Q9JKA9 |
| Dual specificity phosphatase 28 | **50** | 18.7 | 6 | **Q4G0W2** | http://www.uniprot.org/uniprot/Q4G0W2 |
| Hydroxysteroid dehydrogenase-like protein 2 | **63** | 45.5 | 10 | **A4FUZ6** | http://www.uniprot.org/uniprot/A4FUZ6 |
| Islet amyloid polypeptide | **52** | 10 | 5 | **P17716** | http://www.uniprot.org/uniprot/P17716 |
| G-protein coupled receptor-associated sorting protein 1 | **51** | 152.8 | 13 | **Q5U4C1** | http://www.uniprot.org/uniprot/Q5U4C1 |
| Growth/differentiation factor 2 | **52** | 47.9 | 7 | **Q9UK05** | http://www.uniprot.org/uniprot/Q9UK05 |
| Cytosolic purine 5'-nucleotidase | **52** | 65.3 | 10 | **O46411** | http://www.uniprot.org/uniprot/O46411 |
| Vesicle transport protein USE1 | **59** | 30.8 | 16 | **Q9CQ56** | http://www.uniprot.org/uniprot/Q9CQ56 |
| Putative zinc finger protein 137 | **61** | 24.7 | 8 | **P52743** | http://www.uniprot.org/uniprot/P52743 |
| Elongation factor 1-beta | **52** | 25 | 5 | **Q5E983** | http://www.uniprot.org/uniprot/Q5E983 |
| Tektin-4 | **64** | 51.3 | 12 | **Q8WW24** | http://www.uniprot.org/uniprot/Q8WW24 |
| Parathyroid hormone/parathyroid hormone-related peptide receptor | **67** | 66.7 | 9 | **Q1LZF7** | http://www.uniprot.org/uniprot/Q1LZF7 |
| Chorionic somatomammotropin hormone 2 | **50** | 28.2 | 6 | **P19159** | http://www.uniprot.org/uniprot/P19159 |
| Probable tubulin polyglutamylase TTLL1 | **51** | 49.5 | 8 | **Q5PPI9** | http://www.uniprot.org/uniprot/Q5PPI9 |
| Collagen alpha-1(XI) chain (Fragment) | **62** | 89.3 | 10 | **Q28083** | http://www.uniprot.org/uniprot/Q28083 |
| G kinase-anchoring protein 1 | **68** | 42.2 | 10 | **Q5XIG5** | http://www.uniprot.org/uniprot/Q5XIG5 |
| Protein phosphatase Slingshot homolog 1 | **67** | 116.5 | 14 | **Q8WYL5** | http://www.uniprot.org/uniprot/Q8WYL5 |
| Arginine/serine-rich protein 1 | **58** | 33.7 | 8 | **Q9BUV0** | http://www.uniprot.org/uniprot/Q9BUV0 |
| Sorting and assembly machinery component 50 homolog | **50** | 52.2 | 6 | **Q8BGH2** | http://www.uniprot.org/uniprot/Q8BGH2 |
| Cystatin-B | **57** | 11.2 | 4 | **P25417** | http://www.uniprot.org/uniprot/P25417 |
| Desmin | **61** | 53.6 | 13 | **P17661** | http://www.uniprot.org/uniprot/P17661 |
| Transmembrane inner ear expressed protein | **52** | 53.6 | 6 | **Q8K467** | http://www.uniprot.org/uniprot/Q8K467 |
| Putative uncharacterized protein encoded by CRHR1-IT1 | **50** | 17.2 | 6 | **Q96LR1** | http://www.uniprot.org/uniprot/Q96LR1 |
| Oculomedin | **58** | 5.3 | 5 | **Q9Y5M6** | http://www.uniprot.org/uniprot/Q9Y5M6 |
| Protein kish-A | **62** | 8.3 | 5 | **Q148I3** | http://www.uniprot.org/uniprot/Q148I3 |
| Rab GDP dissociation inhibitor beta | **51** | 50.8 | 6 | **O97556** | http://www.uniprot.org/uniprot/O97556 |
| Glutathione S-transferase Mu 6 | **52** | 25.8 | 8 | **O35660** | http://www.uniprot.org/uniprot/O35660 |
| Hemoglobin subunit beta | **68** | 16.3 | 8 | **P02073** | http://www.uniprot.org/uniprot/P02073 |
| Zinc finger and SCAN domain-containing protein 5A | **62** | 56.9 | 11 | **Q9BUG6** | http://www.uniprot.org/uniprot/Q9BUG6 |
| Ras-related protein Rab-36 | **53** | 36.8 | 7 | **O95755** | http://www.uniprot.org/uniprot/O95755 |
| 39S ribosomal protein L50, mitochondrial | **54** | 18.3 | 9 | **Q8VDT9** | http://www.uniprot.org/uniprot/Q8VDT9 |
| NAD-dependent protein deacetylase sirtuin-7 | **51** | 45.7 | 9 | **Q8BKJ9** | http://www.uniprot.org/uniprot/Q8BKJ9 |
| Ras-related protein Rab-36 | **57** | 36.8 | 12 | **O95755** | http://www.uniprot.org/uniprot/O95755 |
| Putative uncharacterized protein encoded by LINC01546 | **62** | 7.3 | 5 | **A6NGU7** | http://www.uniprot.org/uniprot/A6NGU7 |
| Hemoglobin subunit beta | **51** | 16.3 | 7 | **P02073** | http://www.uniprot.org/uniprot/P02073 |
| 3-oxo-5-beta-steroid 4-dehydrogenase | **50** | 37.7 | 8 | **P51857** | http://www.uniprot.org/uniprot/P51857 |
| Synaptosomal-associated protein 29 | **56** | 29.1 | 7 | **Q9Z2P6** | http://www.uniprot.org/uniprot/Q9Z2P6 |
| Zinc finger protein 624 | **52** | 102.5 | 10 | **Q9P2J8** | http://www.uniprot.org/uniprot/Q9P2J8 |
| Ubiquitin carboxyl-terminal hydrolase 48 | **51** | 120.7 | 11 | **Q76LT8** | http://www.uniprot.org/uniprot/Q76LT8 |
| Cholecystokinin | **56** | 12.7 | 4 | **P23362** | http://www.uniprot.org/uniprot/P23362 |
