# Supplementary material for: Urinary proteome of dogs with kidney injury during babesiosis
Source: BMC Vet Res. 2019 Dec 4;15:439. doi: 10.1186/s12917-019-2194-0 (PMC6894246; doi:10.1186/s12917-019-2194-0)
Supplement: Supplementary file 3 — Additional file 3 Table S3. Common proteins for babesiosis and healthy dogs. [file 12917_2019_2194_MOESM3_ESM.docx]

Table S3. List of common proteins identified in the urine of dogs with babesiosis and healthy dogs by MALDI-TOF/TOF.

| Nr | Accession ^a^ | Protein name | UniProt link |
| --- | --- | --- | --- |
| 1 | Q5XFN2 | DESMIN | http://www.uniprot.org/uniprot/Q5XFN2 |
| 2 | Q28298 | Ribosome-binding protein 1 | http://www.uniprot.org/uniprot/Q28298 |
| 3 | P79244 | 60S ribosomal protein L37 | http://www.uniprot.org/uniprot/P79244 |
| 4 | Q5E983 | Elongation factor 1-beta | http://www.uniprot.org/uniprot/Q5E983 |
| 5 | P49822 | Serum albumin | http://www.uniprot.org/uniprot/P49822 |
| 6 | Q8IZC7 | Zinc finger protein 101 | http://www.uniprot.org/uniprot/Q8IZC7 |
| 7 | Q3T035 | Actin-related protein 2/3 complex subunit 3 | http://www.uniprot.org/uniprot/Q3T035 |
| 8 | Q9BUG6 | Zinc finger and SCAN domain-containing protein 5A | http://www.uniprot.org/uniprot/Q9BUG6 |
| 9 | Q9P2J8 | Zinc finger protein 624 | http://www.uniprot.org/uniprot/Q9P2J8 |
| 10 | P12672 | 43 kDa receptor-associated protein of the synapse | http://www.uniprot.org/uniprot/P12672 |
| 11 | Q9DBC3 | Cap-specific mRNA (nucleoside-2'-O-)-methyltransferase 1 | http://www.uniprot.org/uniprot/Q9DBC3 |
| 12 | Q9UJ72 | Annexin A10 | http://www.uniprot.org/uniprot/Q9UJ72 |
| 13 | P35831 | Tyrosine-protein phosphatase non-receptor type 12 | http://www.uniprot.org/uniprot/P35831 |
| 14 | A7MBG3 | Calcium/calmodulin-dependent protein kinase II inhibitor 1 | http://www.uniprot.org/uniprot/A7MBG3 |
| 15 | Q3SZV0 | Tetratricopeptide repeat protein 36 | http://www.uniprot.org/uniprot/Q3SZV0 |
| 16 | D2H6Z0 | E3 ubiquitin-protein ligase RNF152 | http://www.uniprot.org/uniprot/D2H6Z0 |
| 17 | Q4G0W2 | Dual specificity phosphatase 28 | http://www.uniprot.org/uniprot/Q4G0W2 |
| 18 | Q4R517 | Succinyl-CoA ligase [ADP-forming] subunit beta, mitochondrial | http://www.uniprot.org/uniprot/Q4R517 |
| 19 | Q58DV5 | 39S ribosomal protein L30, mitochondrial | http://www.uniprot.org/uniprot/Q58DV5 |
| 20 | Q9UI09 | NADH dehydrogenase [ubiquinone] 1 alpha subcomplex subunit 12 | http://www.uniprot.org/uniprot/Q9UI09 |
| 21 | Q8WW24 | Tektin-4 | http://www.uniprot.org/uniprot/Q8WW24 |
| 22 | P16859 | Natriuretic peptides B | http://www.uniprot.org/uniprot/P16859 |
| 23 | Q5XIG5 | G kinase-anchoring protein 1 | http://www.uniprot.org/uniprot/Q5XIG5 |
| 24 | Q5XIG5 | G kinase-anchoring protein 1 | http://www.uniprot.org/uniprot/Q5XIG5 |
| 25 | Q8N6M0 | OTU domain-containing protein 6B | http://www.uniprot.org/uniprot/Q8N6M0 |
| 26 | Q96LR1 | Putative uncharacterized protein encoded by CRHR1-IT1 | http://www.uniprot.org/uniprot/Q96LR1 |
| 27 | Q08DJ7 | Protein AAR2 homolog | http://www.uniprot.org/uniprot/Q08DJ7 |
| 28 | Q4R5F5 | Interferon-induced protein with tetratricopeptide repeats 1 | http://www.uniprot.org/uniprot/Q4R5F5 |
| 29 | O14746 | Telomerase reverse transcriptase | http://www.uniprot.org/uniprot/O14746 |
| 30 | Q9BUV0 | Arginine/serine-rich protein 1 | http://www.uniprot.org/uniprot/Q9BUV0 |
